# Comparative Transcriptome Analyses of Gayal *(Bos frontalis)*, Yak *(Bos grunniens)*, and Cattle *(Bos taurus)* Reveal the High-Altitude Adaptation

**DOI:** 10.3389/fgene.2021.778788

**Published:** 2022-01-11

**Authors:** Jun Ma, Tianliu Zhang, Wenxiang Wang, Yan Chen, Wentao Cai, Bo Zhu, Lingyang Xu, Huijiang Gao, Lupei Zhang, Junya Li, Xue Gao

**Affiliations:** Laboratory of Molecular Biology and Bovine Breeding, Institute of Animal Science, Chinese Academy of Agricultural Sciences, Beijing, China

**Keywords:** gayal, yak, differentially expressed genes, co-expression, high-altitude adaptation, hypoxia

## Abstract

Gayal and yak are well adapted to their local high-altitude environments, yet the transcriptional regulation difference of the plateau environment among them remains obscure. Herein, cross-tissue and cross-species comparative transcriptome analyses were performed for the six hypoxia-sensitive tissues from gayal, yak, and cattle. Gene expression profiles for all single-copy orthologous genes showed tissue-specific expression patterns. By differential expression analysis, we identified 3,020 and 1,995 differentially expressed genes (DEGs) in at least one tissue of gayal *vs.* cattle and yak *vs.* cattle, respectively. Notably, we found that the adaptability of the gayal to the alpine canyon environment is highly similar to the yak living in the Qinghai-Tibet Plateau, such as promoting red blood cell development, angiogenesis, reducing blood coagulation, immune system activation, and energy metabolism shifts from fatty acid β-oxidation to glycolysis. By further analyzing the common and unique DEGs in the six tissues, we also found that numerous expressed regulatory genes related to these functions are unique in the gayal and yak, which may play important roles in adapting to the corresponding high-altitude environment. Combined with WGCNA analysis, we found that *UQCRC1* and *COX5A* are the shared differentially expressed hub genes related to the energy supply of myocardial contraction in the heart-related modules of gayal and yak, and *CAPS* is a shared differential hub gene among the hub genes of the lung-related module, which is related to pulmonary artery smooth muscle contraction. Additionally, *EDN3* is the unique differentially expressed hub gene related to the tracheal epithelium and pulmonary vasoconstriction in the lung of gayal. *CHRM2* is a unique differentially expressed hub gene that was identified in the heart of yak, which has an important role in the autonomous regulation of the heart. These results provide a basis for further understanding the complex transcriptome expression pattern and the regulatory mechanism of high-altitude domestication of gayal and yak.

## Introduction

Species living at high altitudes are exposed to strict selection pressures and physiological challenges owing to harsh environmental conditions, such as thin air, cold temperatures, ultraviolet exposure, and low pressure (Miao et al., 2015). Despite the harsh conditions, numerous species including Tibetan pigs (Jia et al., 2016; Zhang B. et al., 2017), Tibetan sheep (Zhang et al., 2013), Tibetan chickens (Gou et al., 2014), and yak (Qiu et al., 2012; Qi et al., 2019) have evolved unique physiological characteristics, such as superior blood oxygen transport system and high metabolic efficiency, to adapt to the harsh living pressure (Lan et al., 2018), as have the native Tibetan people (Ge et al., 2012; Tashi et al., 2014). At present, published studies have identified *EPAS1*, *EGLN1,* and *PPARA* genes that play important roles in high-altitude adaptation (Haasl and Payseur, 2016; Heinrich et al., 2019). Exploring the molecular mechanisms underlying hypoxia adaptation has long garnered attention.

Gayal, also known as Drung cattle (*Bos frontalis*) (Winter et al., 1986; Uzzaman et al., 2014), is a unique semi-wild cattle breed, mainly found in the typical alpine valleys and subtropical rain forests in the Drung and Nujiang river basins of Yunnan Province, southwestern China. Studies have shown that the level of hemoglobin in gayal, as well as the number of red blood cells and white blood cells, are equivalent to those of yak, which is the physiological basis for the ability of gayal to resist invasion by bacteria, viruses, and parasites in the alpine environment (Tian et al., 1998). Yak (*Bos grunniens*), a native mammal on the Qinghai-Tibet Plateau and its adjacent regions, provides meat and other necessities for Tibetans. Compared with lowland cattle, yaks have a larger alveolar area per unit area, thinner alveolar spacing, thinner gas-blood barrier (Wei and Yu, 2008), and stronger expression of some genes related to oxygen supply as well as defense system under hypoxia pressure (Wang et al., 2016). Therefore, a thorough understanding of the genetic basis of the physiological characteristics of gayal and yak will provide insight into their adaptation to the high-altitude environment.

With recent rapid progress in next-generation genome sequencing (NGS) technologies, high-throughput RNA-Sequencing (RNA-seq) technology represents a powerful and cost-efficient approach to explore species domestication, breeding, and genetic molecular mechanisms in organisms (Salleh et al., 2017; Keren et al., 2018). RNA-seq can detect and quantify gene expression with digital measurements, which are especially sensitive for low-expressed genes (Mortazavi et al., 2008). In addition, it is also used to improve gene annotation, discover novel genes or transcripts, and survey alternative splicing (AS) events at single-nucleotide resolution (Sultan et al., 2008; Ozsolak and Milos, 2011). Thus far, transcriptome studies on gayal and yak have been widely conducted. The muscle transcriptome analysis of Indian Mithun showed that hub genes including *MTMR3, CUX1, LONRF3, PLXNB2, KMT2C, ZRSR2Y, PRR14, USP9Y, SLMAP,* and *KANSL2* might contribute to muscle growth (Mukherjee et al., 2020). Transcriptomic analysis of yaks living at different altitudes indicates PI3K-Akt, HIF-1, focal adhesion, and ECM–receptor interaction pathway were significantly enriched, and the *EPAS1* expression increases with altitude (Qi et al., 2019). Comparative transcriptome analysis of four organs (heart, kidney, liver, and lung) between yak and cattle revealed that DEGs associated with the blood supply system, modulation of cardiac contractility, vascular smooth muscle proliferation, and the glutamate receptor system probably play an important role in yak adaptation to hypoxia environments (Wang et al., 2016). However, gayal is a species that is well adapted to their local alpine valley environment, and few transcriptomic studies focusing on regulation analysis of gayal in adaptation to the local high-altitude environment have so far not been reported. In addition, studies have found specific adaptive genes and mechanisms are distinct between species and populations (Heinrich et al., 2019; Pamenter et al., 2020). Therefore, we seek to understand how changes in altitude affect the transcriptional regulation of gayal, and whether the relevant transcriptional regulation mechanism is the same as that of yak, which shares a bovine subfamily.

In this study, we characterized gene expression profiles using RNA-seq data from six major tissues including heart, lung, liver, kidney, spleen, and muscle among gayal, yak, and cattle. We performed a comparative transcriptome analysis of six tissues in gayal *vs.* cattle and yak *vs.* cattle, respectively, and identified candidate DEGs related to adaptability to the plateau environment, as well as analyzed their functions and expression patterns. In addition, weighted gene co-expression network analysis (WGCNA) was further used to uncover the hub genes that regulate tissue function in high-altitude adaptation. The results could lay a foundation for further explorations of the genetic changes underlying hilly adaptations in subtropical mammals.

## Materials and Methods

### Sample Collection

Six tissues (heart, kidney, liver, lung, skeletal muscle, and spleen) were collected from three adult female gayal living in the semi-wild preserved field at an altitude of 2,300–3,500 m in the Drung and Nujiang river basins. All tissue samples were taken and immediately snap-frozen in liquid nitrogen. In addition, the corresponding tissue transcriptome data of yak and cattle were retrieved from a previous publication (Tang et al., 2017) from the GEO databases under the accession numbers GSE93855 and GSE77020 (Supplementary Table S1). In addition, one gayal multi-tissue transcriptome data that we previously published was also included in this study to expand the sample size, which was stored in National Genomics Data Center under the accession code PRJCA002143. An overview of the samples used and the associated statistics are provided in Supplementary Table S1.

### RNA Extraction, Library Construction, and Transcriptome Sequencing

Total RNA was isolated and purified with the TRIzol reagent (Life Technologies, Carlsbad, United States) according to the manufacturer’s protocols. RNA purity was qualified using NanoDrop ND 2000 spectrophotometer at 260 and 280 nm (Thermo Fisher Scientific, Wilmington, United States), and RNA integrity was assessed using Agilent 2,100 Bioanalyzer (Agilent Technologies, Palo Alto, United States). The OD260/280 ratios of all samples were greater than 1.8, and the RNA integrity number (RIN) value of >7.0. Then, mRNA was broken down into small fragments using a magnesium RNA cleavage module (NEB, Ipswich, United States). Random primers and related reverse transcriptase were used to synthesize the first-strand complementary DNA (cDNA). Subsequently, the second-strand cDNA was synthesized. The average insert size for the final cDNA library was 300 ± 50 bp. Finally, we conducted the 2 × 150 bp paired-end sequencing (PE150) on an Illumina Novaseq™ 6,000 (LC-Bio Technology CO., Ltd., Hangzhou, China) following the supplier’s recommended protocol.

### Identification of Orthologous Genes

To identify orthologous genes among gayal, yak and cattle, we first downloaded protein sequence data of yak and cattle from the Ensemble genome browser 104 (http://asia.ensembl.org/index.html) and combined with the protein sequence data of the gayal reference genome assembled by ourselves (the accession code of National Genomics Data Center is PRJCA004132) for downstream analysis. We used ORTHOFINDER v2.2.6 (Emms and Kelly, 2015) software to delimit orthologous groups, in which all predicted protein sequences were compared using a BLAST all-against-all search (Camacho et al., 2009). The single-copy genes, duplicated genes, and species-specific genes were extracted from the ORTHOFINDER output, respectively. Among them, the 1:1:1 homologous genes of the three species were defined as the conserved single-copy homologous genes of each species, which were retained for the following analysis.

### Data Quality Control, Processing, and Normalization of Gene Expression Levels

To ensure the accuracy of subsequent biological information analysis, the quality of the raw data was first checked using FastQC (http://www.bioinformatics.babraham.ac.uk/projects/fastqc/). According to the evaluation results of raw reads, the software FASTP (https://github.com/OpenGene/fastp) with default parameters was used to remove adapters, reads with a high proportion of unknown nucleotides, and low-quality sequences (≤Q20) to obtain high-quality clean reads. To avoid biases due to sequence divergence across bovine subfamily genomes, we downloaded yak (BosGru_v2.0, http://ftp.ensembl.org/pub/release-104/fasta/bos_mutus/) and cattle (ARS_UCD1_2, http://ftp.ensem -bl.org/pub/release-104/fasta/bos_taurus/) reference genomes from Ensemble database, and retrieved gayal (Drung_v1.2) reference genomes from National Genomics Data Center (NGDC) (https://bigd.big.ac.cn/). Then, the clean reads for each sample were mapped to the corresponding reference genome using HISAT2 software (Pertea et al., 2016). FeatureCount from the Subread package (Version 2.0.0) (Liao et al., 2014) was applied to calculate the numbers of reads mapped to each gene. Read pairs were counted only if they were uniquely mapped and properly paired. The gene expression level estimates may be biased among species due to factors such as the size of mRNA transcript and the qualities of different genome annotations (Blake et al., 2020). To avoid these issues, we used a custom script to only retain reads that mapped to the 1:1 orthologous which can be used for each of the three genomes (Blake et al., 2020). Taking into account the difference in sequencing depth of samples from different sequencing platforms, we first filtered out genes with low expression, and only keep genes with expression level CPM> 1 in at least half of the samples (Blake et al., 2020). We normalized the raw read counts using the weighted trimmed mean of M-values algorithm (TMM) in the edgeR package (Robinson et al., 2010). Then scaling factors were used to scale the expression levels of all orthologous. The normalized expression data for the trimmed orthologous were used for subsequent gene expression analysis.

### Gene Expression Profile Analysis

After performing normalization, we obtained the TMM-normalized log_2_-transformed CPM values for each gene. The principal component analysis (PCA) was performed with the prcomp function in R using normalized log_2_-transformed CPM values. The hierarchical clustering of Pearson’s correlation coefficients for each pair of samples was performed using the complete-linkage agglomerative method on the correlation distance matrix and generated symmetrical heatmap plot using the R gplots package.

### Differential Expression Pattern Analysis

To characterize the high-altitude adaptive gene expression pattern of gayal and yak, we performed differential expressed analyses among heart, kidney, liver, lung, skeletal muscle, and spleen tissues in gayal *vs.* cattle group and yak *vs.* cattle group by using R package edgeR (Robinson et al., 2010). Genes with Benjamini Hochberg false discovery rate (FDR) values less than 0.05 and fold change more than 2 were identified as DEGs. If the log_2_ fold changes more than 1 were defined as up-regulated DEGs, or less than -1 were defined as down-regulated. We performed Gene Ontology (GO) function annotation and KEGG pathway enrichment analysis for DEGs by the Database for Annotation, Visualization and Integrated Discovery (DAVID) v6.8 using the *Bos taurus* genome as the background set (Huang Da et al., 2009). The significantly enriched GO and KEGG terms using a 0.05 cutoff for the *p*-value with Benjamini-Hochberg test adjustment. The jvenn and UpSetR R package (version 1.4.0) were used to identify and visualize the DEGs shared by two comparison groups across the multiple tissues and between two comparison groups in the same tissue, respectively (Conway et al., 2017). In addition, to better understand the hypoxia adaptation of species, we also collected a set of known or potential high-altitude adaptation candidate genes from previously published literature (Zhang et al., 2014; Wang et al., 2016; Peng et al., 2017).

### Construction of Gene Co-Expression Network

The adaptation of species to the hypoxia environment is a complex biological process, which involves the coordinated regulation of multiple genes. WGCNA is a feasible method to clarify the regulatory relationship between genes and identify important hub genes based on gene expression profiles to explore the mechanism of hypoxia adaptation (Wang et al., 2021). Therefore, to further identify the relevant regulatory gene modules related to the functions of each tissue and explore the core driver genes, we used the CPM values of filtered genes to perform WGCNA analysis on three species, respectively (Langfelder and Horvath, 2008). The hierarchical cluster analysis on all tissues was carried out using the hclust function. The soft-power threshold beta was determined by the function “sft$powerEstimate” (Chen et al., 2019). The minimal gene module size was set to 30 to obtain appropriate modules, and the threshold to merge similar modules was set to 0.25. Gene modules were detected based on the TOM matrix. The correlations between modules eigengene and tissues were investigated using the Pearson correlation, and only modules with correlation coefficients >0.7 and *p* < 0.001 were considered tissue-relevant modules (Touma et al., 2016). Here, we designate the highest top 5% of genes as the hub genes based on the intramodular connectivity in each functional module, which is calculated by summing the connection strengths with other intramodular genes (Yang et al., 2014). Functional enrichment analyses for tissue-related module genes were performed using DAVID v6.8 using the *Bos taurus* genome as the background set, and the *p*-values were corrected for multiple testing using Benjamini-Hochberg correction (Huang Da et al., 2009).

## Results

### Summary Statistics of RNA-Seq Data

The transcriptome data of six tissues (heart, kidney, liver, lung, skeletal muscle, and spleen) from four gayals, three yaks, and three cattle were used in this study (Supplementary Table S1). One gayal heart sample (gayal3_heart) was excluded due to low sequencing quality. In total, ∼327.55 G clean data has been generated after removing adapter-embedded or low-quality reads. We calculated Q20 (99% base call accuracy), Q30 (99.9% base call accuracy), and GC-content of the clean data. The average values of Q20 and Q30 in the samples were 97.04% (ranging from 94.60 to 98.47%) and 92.38% (ranging from 89.39 to 95.43%), respectively (Supplementary Table S2). The average base GC content was 50.80%, ranging from 47.69 to 55.03%, indicating that the base composition and quality of the sequencing data were qualified. Then the high-quality reads of each sample were mapped to the corresponding reference genome (i.e., Drung_v1.2, BosGru_v2.0, ARS_UCD1_2) using the HISAT2 software (Pertea et al., 2016). The average reads mapping rates in gayal, yak, and cattle were 86.35, 93.60, and 95.58%, respectively. Detailed statistics and mapping information for each sample were summarized in Supplementary Table S2.

### Ortholog Identification and Gene Expression Levels Normalization

To reduce the effect of chromosome number difference (*Bos frontalis*, 2n = 58; and *Bos grunniens*, 2n = 60; *Bos taurus*, 2n = 60) and the qualities of different genome annotations among three bovine species, we first used the blast function of ORTHOFINDER2 (Emms and Kelly, 2015) to identify orthologous genes among the gayal, yak and cattle. A total of 19,174 protein orthogroups were identified. Among them, only 6,371 orthogroups were high-confidence single-copy orthogroups of three species (Supplementary Table S3). Then the corresponding gene names were extracted by a custom script and retained reads that mapped to the single-copy homologous genes of three species genome, and the ensemble gene names of cattle were uniformly used for subsequent analysis. In the 6,371 single-copy orthologous gene expression datasets, genes with low expression (CPM <1) in more than half of the samples were further excluded, and 4,712 genes were obtained (Blake et al., 2020). Then we normalized 4,712 single-copy orthologous genes using the weighted TMM of edgeR (Hao et al., 2019).

### Gene Expression Profiles Across Tissues and Bovine Species

**FIGURE 1:**
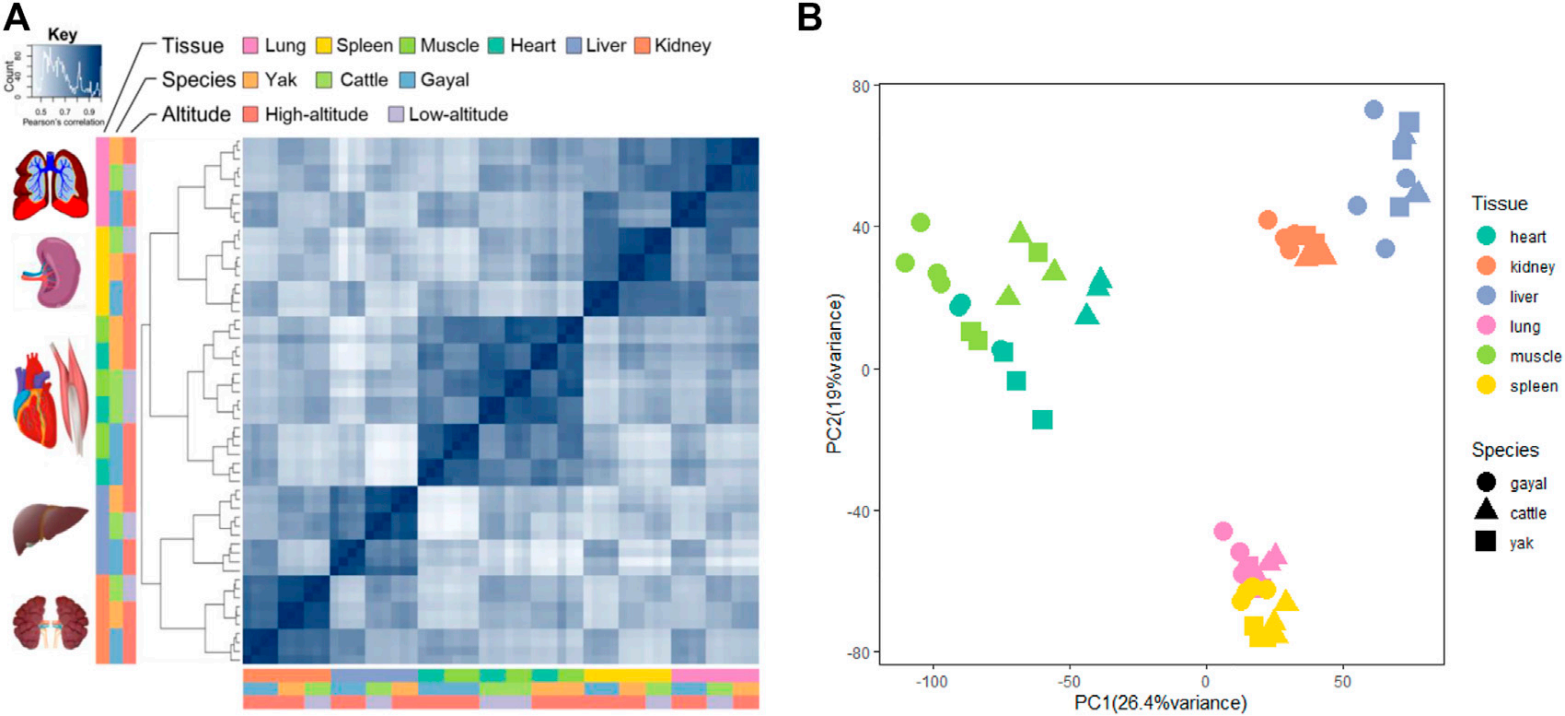
Gene expression patterns across the three bovine species: yak, gayal, and cattle. **(A)** symmetrical heatmap of Pearson’s correlation coefficients between all pairs of samples across all genes. The key is hierarchically clustered (complete method, correlation distance). **(B)** PCA of the log-transformed normalized expression levels of all orthologs across all species and tissues. Species are represented by point shape; tissues are represented by point color.

To investigate broad gene expression patterns in multiple tissues across different bovine species, we performed hierarchical cluster analysis based on the gene expression of six tissues among gayal, yak, and cattle. As shown in Figure 1A, the tissue-specific expression pattern of lung, liver, spleen, and kidney shows that the same tissues of different bovine species gather earlier than different tissues of the same bovine species. The result indicated that tissue differentiation may precede species differentiation, which was consistent with previous studies (Necsulea and Kaessmann, 2014; Hao et al., 2019). However, the samples of heart and muscle from each species were more similar than the same tissue between different species (species-specific expression pattern), which reflects striated muscular tissues may have similar expression profiles (Merkin et al., 2012; Tang et al., 2017; Feng et al., 2020) (Figure 1A). In addition, our expression profiles suggested that the differences in global gene expression among tissues were more significant than that among altitudes or the same tissue of different species, which was consistent with the results of previous studies (Necsulea and Kaessmann, 2014; Tang et al., 2017; Hao et al., 2019). Furthermore, principal component analysis (PCA) was performed on the gene expression of 59 samples. The score plot of PCA analysis for six main organs shows that 45.4% of the variance can be explained by the first two principal components, accounting for 26.4 and 19% of the variance, respectively (Figure 1B). In essence, 59 samples were clustered based on similar tissue types, which confirmed that tissue differentiation may precede species differentiation.

### Gene Expression Changes Accompanying High-Altitude Environment

**FIGURE 2:**
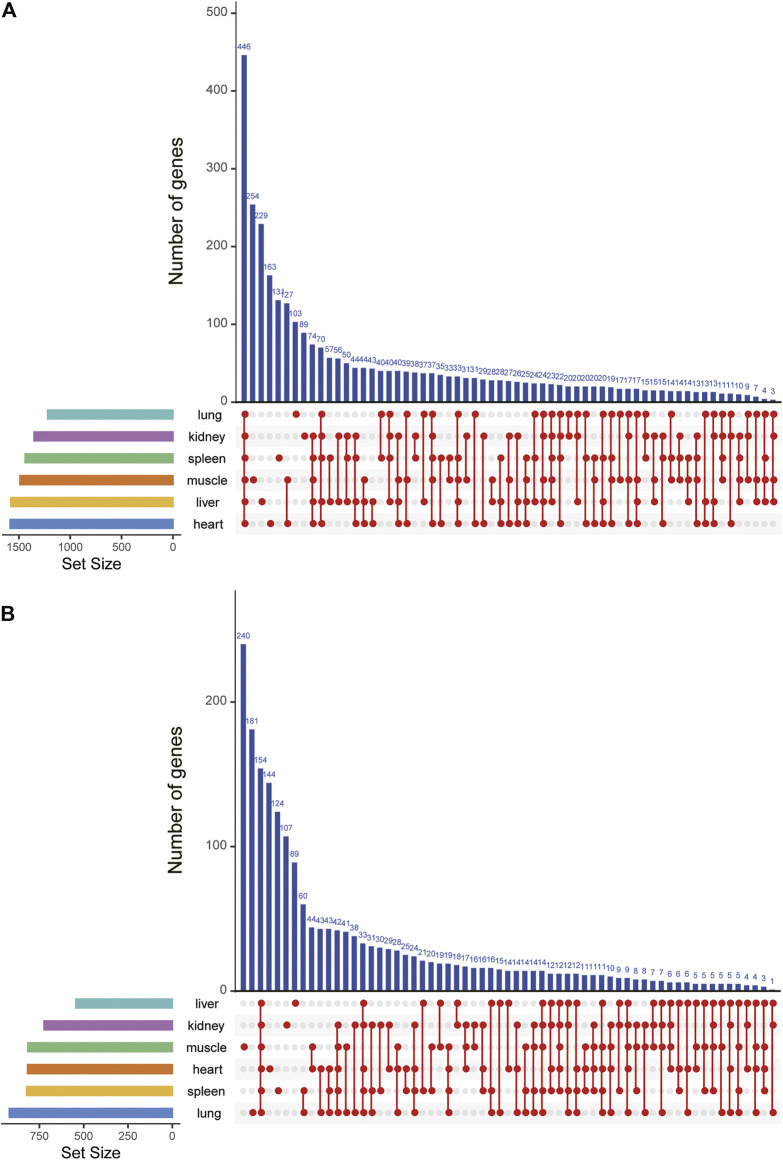
DEGs upset map of all DEGs in six tissues of **(A)** gayal *vs.* cattle group and **(B)** yak *vs.* cattle group.

To detect expression changes of gayal and yak in response to the high-altitude stress, we used edgeR (Robinson et al., 2010) to identify DEGs within each tissue. According to the tissue and species we considered, 1,218 to 1,585 interspecies DEGs were identified in the comparison of six type tissues in gayal and cattle (Table 1). Among the six tissues, the heart has the largest number of DEGs (618 up-regulated and 967 down-regulated genes). By upset analysis of all DEGs in each tissue, 446 DEGs shared in six tissues were identified, and each gene showed consistent up-regulation or down-regulation in all tissues (Figure 2A). In the yak *vs.* cattle group, we identified 1,995 DEGs among six types of tissues. The number of DEGs among tissues ranges from 543 in liver tissue (202 up-regulated and 341 down-regulated genes) to 919 in lung tissue (387 up-regulated and 532 down-regulated genes) (Table 1). There were 154 DEGs with consistent regulation direction shared among the six tissues, of which 64 genes were also present in all tissues of the gayal *vs*. cattle group (Figure 2B).

**TABLE 1:** The number of DEGs identified in six tissues for gayal and yak compared with low-altitude cattle.

Group	Up- or down-regulated	Heart	Liver	Spleen	Lung	Kidney	Muscle
Gayal VS cattle	Up	618	764	599	512	586	560
	Down	967	812	839	706	766	929
	Total	1,585	1,576	1,438	1,218	1,352	1,489
Yak VS cattle	Up	278	202	313	387	279	367
	Down	537	341	508	532	443	446
	Total	815	543	821	919	722	813

**FIGURE 3:**
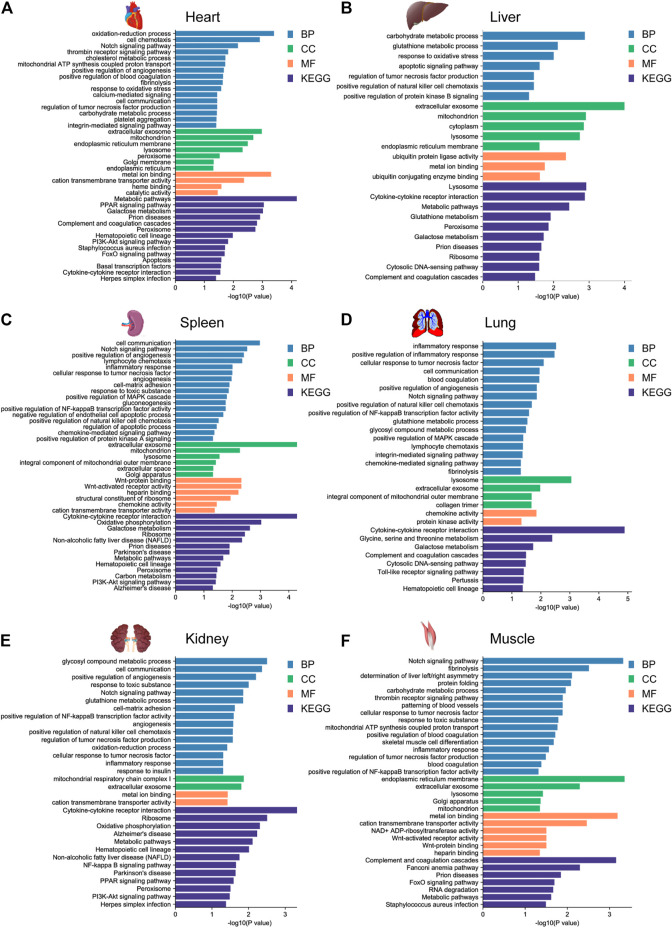
GO functional and KEGG pathway enrichment analysis of DEGs in the **(A)** heart, **(B)** liver, **(C)** spleen, **(D)** lung, **(E)** kidney, and **(F)** muscle for the gayal *vs.* cattle group.

**FIGURE 4:**
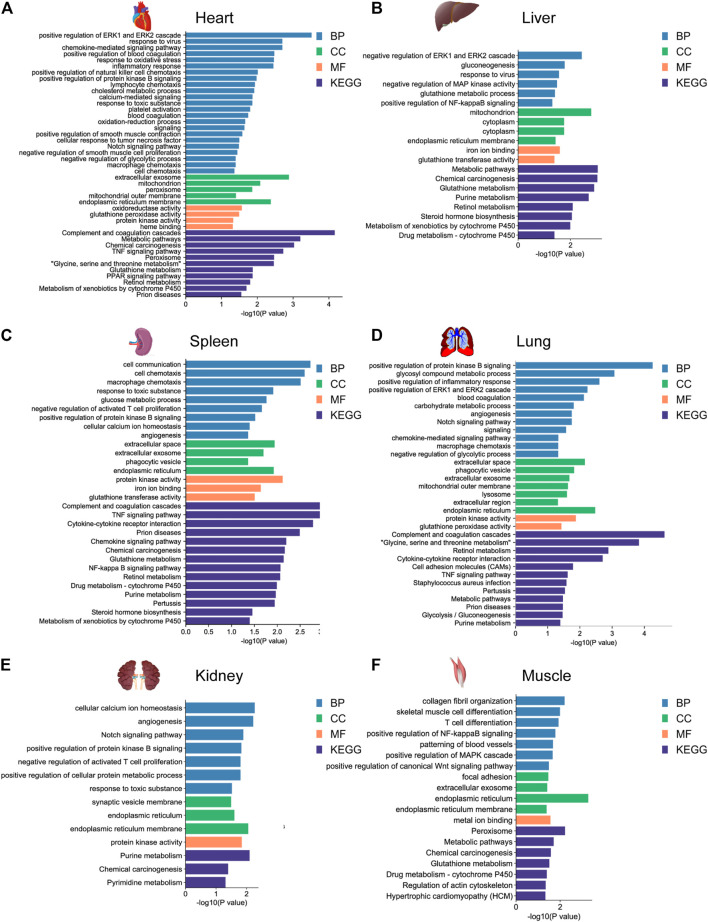
GO functional and KEGG pathway enrichment analysis of DEGs in the **(A)** heart, **(B)** liver, **(C)** spleen, **(D)** lung, **(E)** kidney, and **(F)** muscle for the yak *vs.* cattle group.

To further investigate the potential biological function of DEGs for environmental adaptation in gayal and yak, we performed functional annotation analysis based on the DAVID database. In the gayal *vs.* cattle group, DEGs were annotated in energy metabolism, cardiovascular system, blood coagulation system, and immune system among tissues (Supplementary Table S4). As such, the DEGs of the heart were enriched in the oxidation-reduction process (GO:0055114), positive regulation of angiogenesis (GO:0045766), positive regulation of blood coagulation (GO:0030194). Muscle DEGs were enriched in blood coagulation (GO:0007596), patterning of blood vessels (GO:0001569), skeletal muscle cell differentiation (GO:0035914) (Figure 3). We also observed DEGs in the gayal *vs.* cattle group were significantly enriched in hypoxia response, metabolism, and immune system pathway. For instance, hematopoietic cell lineage (bta04640) was enriched in four tissues (heart, spleen, lung, and kidney) and galactose metabolism (bta00052) was enriched in four tissues (heart, liver, spleen, and lung) (Supplementary Table S4). In the yak *vs.* cattle group, DEGs identified across six tissues pairwise comparisons were also enriched in the cardiovascular system, energy metabolism, and blood coagulation system, which included response to oxidative stress (GO:0006979) and oxidation-reduction process (GO:0055114) in the heart, and patterning of blood vessels (GO:0001569) and positive regulation of canonical wnt signaling pathway (GO:0090263) in the muscle (Figure 4). Similar results were found in the KEGG analysis, pathways related to energy metabolism and the immune system were significantly enriched (Supplementary Table S4).

**FIGURE 5:**
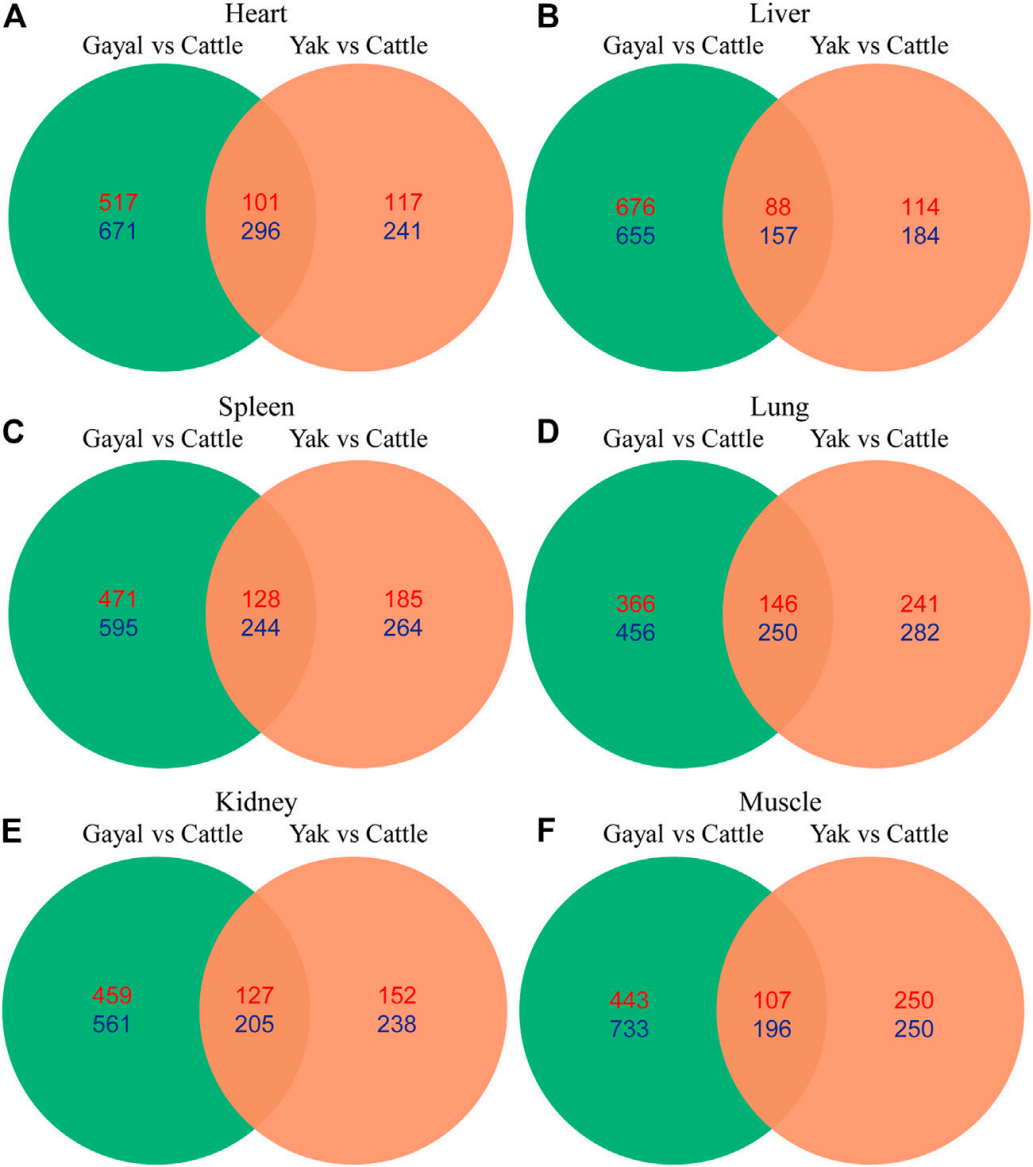
Venn diagram indicating differentially expressed genes that were shared among the two high- and low-altitude pairs in **(A)** heart, **(B)** liver, **(C)** spleen, **(D)** lung, **(E)** kidney, and **(F)** muscle. Numbers in red and blue indicate genes up- and down-regulated in the high-altitude Bovina species relative to low-altitude cattle.

To explore the similarities and differences of expression regulation genes related to environmental adaptability among gayal and yak, we further analyzed the shared and unique DEGs between the gayal *vs.* cattle group and the yak *vs.* cattle group. Among the six tissues of gayal and yak, the heart and lung shared the largest number of DEGs, with 397 (101 up-regulated and 296 down-regulated genes) and 396 genes (146 up-regulated and 250 down-regulated genes), respectively (Figure 5). In addition, we have identified 822–1,331 and 298–500 unique DEGs in the six tissues of gayal and yak compared with lowland cattle, respectively (Figure 5). By functional annotation analysis, we observed the shared DEGs of the heart were enriched in the oxidation-reduction process (GO:0055114), and positive regulation of blood coagulation (GO:0030194). Lung-shared DEGs were involved in platelet activation (GO:0030168), and the intrinsic apoptotic signaling pathway in response to DNA damage (GO:0008630). Liver-shared DEGs were enriched in innate immune response (GO:0045087). Spleen-shared DEGs were enriched in chemotaxis (GO:0006935). And the shared DEGs in the muscle were enriched in skeletal muscle cell differentiation (GO:0035914) (Supplementary Table S4).

As for the unique DEGs of the gayal *vs.* cattle group, we found that five unique DEGs in the heart related to apoptosis (bta04210), include *CASP9, ENDOG, AIFM1, CASP6, TNFSF10, RIPK1, FASLG, and TNF*. Seven unique genes (*BLOC1S6, SERPINE2, TFPI2, HPS4, SERPING1, GP1BA, and TFPI*) are associated with blood coagulation (GO:0007596) in the lung. Spleen-unique DEGs were enriched in lymphocyte chemotaxis (GO:0048247) and inflammatory response (GO:0006954). Furthermore, we found that positive regulation of angiogenesis (GO:0045766) was significantly enriched in specific genes in the lung, kidney, and spleen; hematopoietic cell lineage (bta04640) was also significantly enriched in the unique genes of the heart, spleen, lung, and kidney. In addition, we found that the unique DEGs in the heart, liver, and spleen were also related to galactose metabolism (bta00052). These unique DEGs may play an important role in the adaptation of gayal to the alpine and valley environment (Supplementary Table S4). Among the unique DEGs of the yak *vs.* cattle group, we found that four genes (*CD40LG, SRF, F2R, and F5*) related to platelet activation (GO:0030168) in the heart. Blood coagulation (GO:0007596) related genes (*PROCR, THBD, F10, PROS1, F3, and KNG1*) were also significantly enriched in the lung. The unique genes in the spleen were enriched in immune-related items, such as the NOD-like receptor signaling pathway (bta04621), and TNF signaling pathway (bta04668). In addition, cell adhesion molecules (CAMs) (bta04514) were uniquely enriched in the heart and lung of yak, which was reported as the intensity of hypoxia increases lead to marked reduced cell adhesion (Kaiser et al., 2012) (Supplementary Table S4).

### Expression Regulation of High-Altitude Related Genes in the DEGs

To further establish the hypoxia adaptation of gayal and yak, we collected a candidate gene set with known or potential functions in hypoxia responses from previously published literature (Zhang et al., 2014; Peng et al., 2017). Of those, 295 and 187 genes were DEGs in at least one tissue of gayal and yak, respectively (Supplementary Table S5). Among which the heart has the largest number of high-altitude related genes in both comparison groups DEGs (149 in gayal *vs.* cattle group and 85 genes in yak *vs.* cattle group). Notably, in the altitude-related gene set, we found that several positively selected genes related to hypoxia response in Tibetans were differentially expressed in two comparison groups. For example, *EGLN1*, also known as *PHD2*, as a known target gene of the hypoxia-sensing pathway (Haasl and Payseur, 2016; Heinrich et al., 2019; Storz, 2021), showed a consistent down-regulation across all tissues of yak and gayal compared with lowland cattle, but the expression level and pattern vary in different tissues. *HFE* (Homeostatic Iron Regulator), a gene involved in indirectly regulating iron balance in animals via reducing transferrin-mediated iron uptake (Salter-Cid et al., 1999), was uniquely down-regulated in all tissues of gayal except for kidney compared with the lowland cattle. Additionally, *HOXB6*, a gene associated with the regulation of the erythropoietic system, was particularly up-regulated in the spleen and kidney of the yak (Zimmermann and Rich, 1997).

### Tissue-specific Module Detection and Hub Genes Identification

**FIGURE 6:**
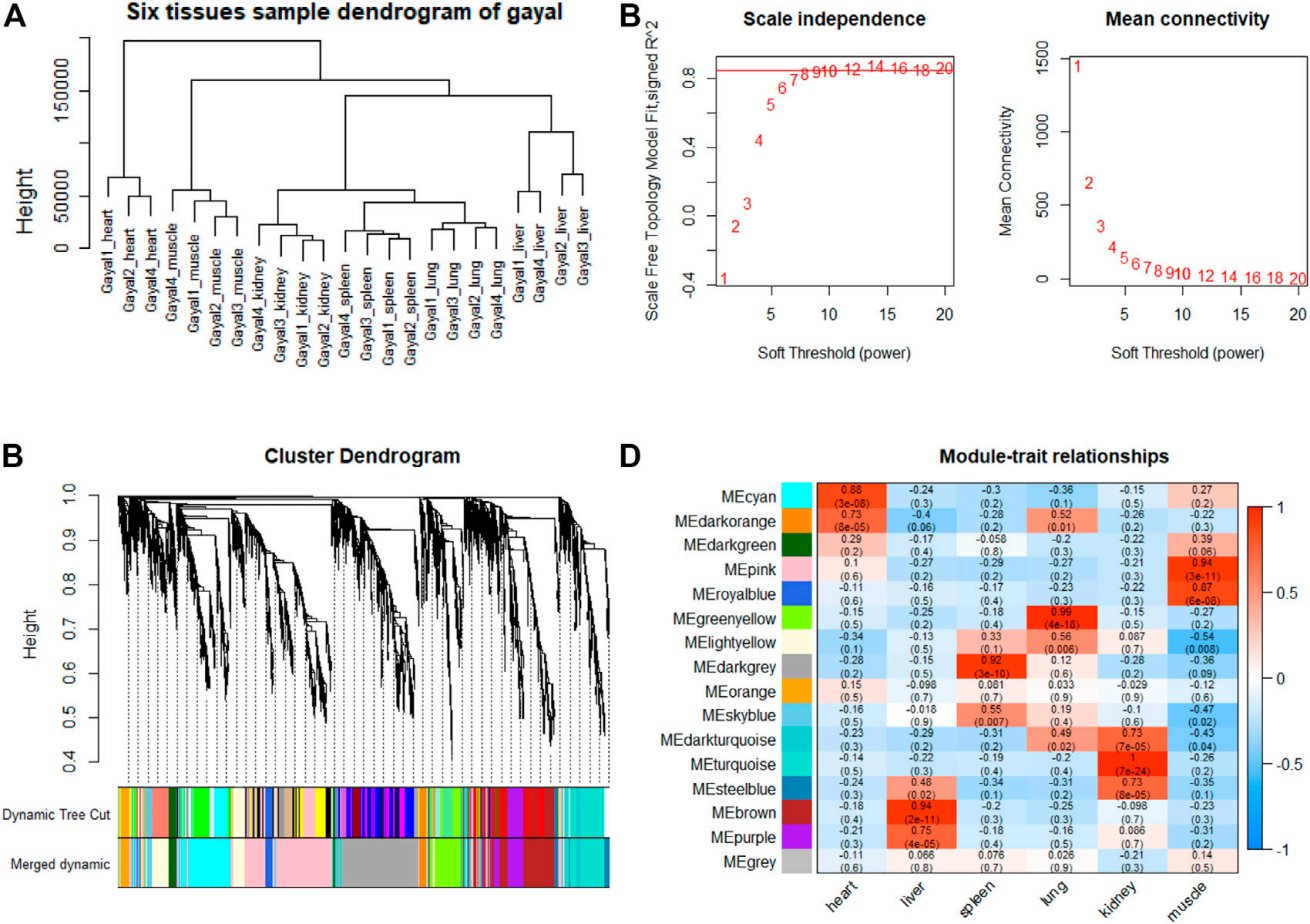
WGCNA analysis of gayal tissue samples. **(A)** Hcluster diagram of gayal tissue samples. **(B)** Plot of scale-free topology and mean connectivity in regard to soft-thresholding power for samples. **(C)** Hierarchical clustering tree (dendrogram) of genes based on co-expression network analysis. **(D)** Heatmap of the correlation between the module eigengenes and tissues of gayal.

**FIGURE 7:**
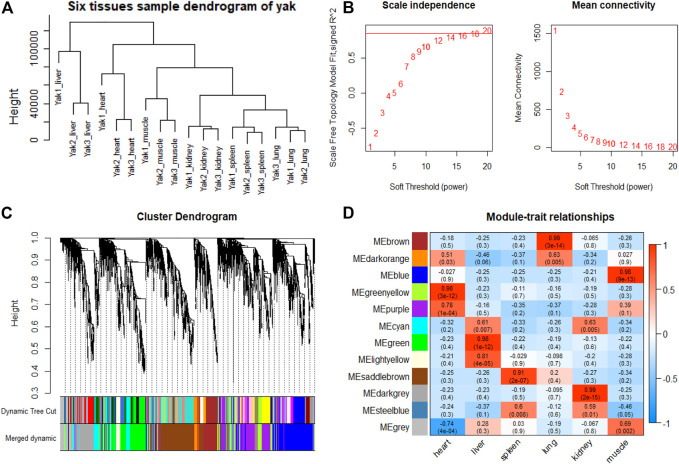
WGCNA analysis of yak tissue samples. **(A)** Hcluster diagram of yak tissue samples. **(B)** Plot of scale-free topology and mean connectivity in regard to soft-thresholding power for samples. **(C)** Hierarchical clustering tree (dendrogram) of genes based on co-expression network analysis. **(D)** Heatmap of the correlation between the module eigengenes and tissues of yak.

**FIGURE 8:**
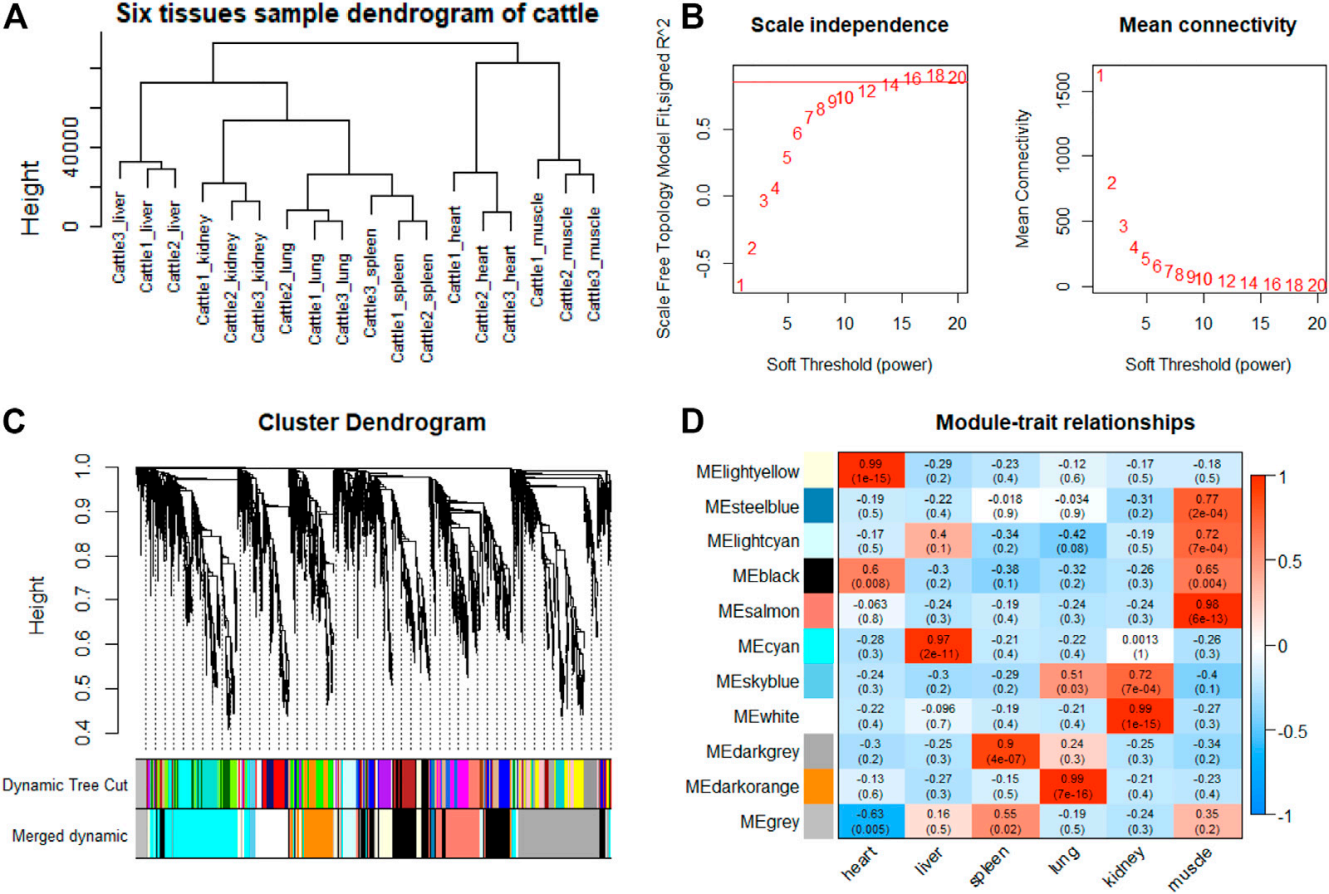
WGCNA analysis of cattle tissue samples. **(A)** Hcluster diagram of cattle tissue samples. **(B)** Plot of scale-free topology and mean connectivity in regard to soft-thresholding power for samples. **(C)** Hierarchical clustering tree (dendrogram) of genes based on co-expression network analysis. **(D)** Heatmap of the correlation between the module eigengenes and tissues of cattle.

To explore genes and co-expression modules with similar expression profiles related to high-altitude adaptation in various tissues, WGCNA analysis was independently performed on the three species based on the CPM values of 4,712 filtered genes. The sample clustering dendrogram of the gayal, yak, and cattle are shown in Figures 6A, 7A, 8A, respectively. In both species, all samples were included in the clusters. The soft-power threshold of 10, 18, and 16 (scale-free *R* ^2^ of 0.85) were selected for further analysis of the gayal, yak, and cattle, respectively (Figures 6B, 7B, 8B). Next, the gene modules were detected based on the topological overlap matrix (TOM), and sixteen modules were detected under the gayal, while ten modules were detected under the yak (Figures 6C, 7C, 8C). To identify tissue-specific modules, we calculated the correlation coefficients between the module and each tissue (Figures 6D, 7D, 8D). Further, GO and KEGG analyses were conducted to investigate the biological function of the genes in each tissue-related module.

Indeed, the relevant modules enriched in six tissues are highly related to the tissue-specific functions. For example, in the tissue-related modules of gayal, the MEcyan module was significantly correlated with heart (r = 0.88, *p* = 3 × 10^–8^), which was enriched in functional annotations such as negative regulation of cardiac muscle cell apoptotic process (GO:0010667), and pathway in MEcyan module were involved in cardiac muscle contraction (bta04260). Lung-related module (MEgreenyellow, r = 0.99, *p* = 4 × 10^–18^) was enriched in HIF-1 signaling pathway (bta04066) (Supplementary Table S6). Similar results were found in the yak, MEgreenyellow was the most significantly correlated with the heart of yak (r = 0.98, *p* = 3 × 10^–12^), which was enriched in functional annotations such as positive regulation of cardiac muscle cell proliferation (GO:0060045), positive regulation of osteoblast differentiation (GO:0045669), and KEGG including Adrenergic signaling in cardiomyocytes (bta04261). The module of MEbrown was significantly correlated with the lung of yak (r = 0.99, *p* = 3 × 10^–14^), which was involved in response to hypoxia (GO:0001666) (Supplementary Table S6). For low-altitude cattle, the heart-related module (MElightyellow, r = 0.99, *p* = 1 × 10^–15^) was enriched in cardiac right ventricle morphogenesis (GO:0003215), negative regulation of cardiac muscle cell apoptotic process (GO:0010667), and heart development (GO:0007507). Lung-related module (MEdarkorange, r = 0.99, *p* = 7 × 10^–16^) was enriched in lung development (GO:0030324). Additionally, in the tissue-related module of each species, the terms related to bile secretion were found in the liver-specific module, functions related to ion transport and absorption are enriched in the kidney-specific module, and immune and skeletal muscle cell differentiation-related functions were enriched in the spleen-specific and muscle-specific module, respectively (Supplementary Table S6).

Furthermore, genes with the highest intramodular connectivity were selected as hub genes in each tissue-related module in both gayal and yak. We identified 4–51, 6–44, and 2–46 hub genes in six tissue-related modules of gayal, yak, and cattle, respectively (Supplementary Table S7). Among the heart-related modules of the three species, there are fourteen common hub genes between gayal and yak, of which five genes are involved in cardiac contraction (bta04260), including *UQCRC1, UQCRQ, UQCRFS1, UQCR11*, and *COX5A*. Among them, *UQCRC1*, *UQCRQ*, *UQCRFS1*, and *UQCR11* are important subunit of mitochondrial complex Ⅲ (Fernández-Vizarra and Zeviani, 2015). Mitochondria play a key role in cardioprotection and heart contraction energy needed for pumping blood to oxygenate the body organs (Lemieux and Hoppel, 2009). *UQCRC1* directly affects mitochondria function related to cardioprotection (Yi et al., 2020). *COX5A* is the component of the cytochrome c oxidase, which is central to oxidative phosphorylation and ATP generation (Huang et al., 2019). However, no common hub gene was found between gayal and low-altitude cattle, and only four hub genes are shared by yak and cattle, including *KCNJ8*, *MTUS2*, *P4HTM*, and *BTG3*. *KCNJ8* encodes a subunit of an ATP-sensitive potassium channel and regulates potassium inward rectifier, which is related to heart development (Erginel-Unaltuna et al., 1998). There are one (*COX6A2*) and two (*CYC1* and *UQCR10*) non-common hub genes in the heart-related modules of gayal and yak, respectively, which are involved in cardiac contraction. In addition, four hub genes (*HAND2*, *CHRM2*, *EDNRA*, and *ZFPM2*) associated with heart development and regulation were uniquely identified in the greenyellow module of the yak heart. Among these, *CHRM2* was an up-regulated DEG in the yak heart (log_2_ fold changes of 1.14), which plays a fundamental role in autonomic regulation of the heart, such as heart rate recovery after maximal exercise (Hautala et al., 2006). *HAND2* is essential for cardiac morphogenesis, vascular development, and the regulation of angiogenesis (Anderson et al., 2016). *EDNRA* encodes the endothelin 1 (*EDN1*) receptor, a gene involved in the vasoconstriction mechanism (Sharma et al., 2014), as well as closely related to pulmonary hypertension and HIF activity, which is positively selected in Tibetan (Simonson et al., 2010). *ZFPM2*, regulates the activity of GATA family proteins, plays a vital function in cardiac morphogenesis and coronary vascular development (Luo et al., 2020).

Among the hub genes of the lung-related module, six common hub genes were identified among gayal and yak, including *CAPS*, *TSNAXIP1*, *GPRIN2*, *SPEF1*, *BMP3*, *and EFHC1*. Gayal and yak have two (*BMP3* and *SEC14L3*) and three (*BMP3*, *DNAAF1*, and *N4BP3*) hub genes in common with low-altitude cattle respectively. Among them, *BMP3*, a hub gene shared by all three bovine subfamily species, has been reported to play a regulatory role in the morphogenesis and/or function of the human lung (Vukicevic et al., 1994). *CAPS* encodes a calcium-binding protein and plays a role in the regulation of Ca^2+^ transport (Pan et al., 2019), which may be correlated with pulmonary artery smooth muscle contraction (Waypa and Schumacker, 2002). *SEC14L3* is an up-regulated DEG in the gayal lung and plays an important role in maintaining the homeostasis of airway epithelial cells (Shan et al., 2009). *DNAAF1* gene is associated with lung development (Duquesnoy et al., 2009). As for the non-common hub genes in the lung-related module of gayal, we discovered several genes (*EDN3*, *GJA5*, and *CCDC39*) related to the tracheal epithelium and pulmonary vasoconstriction. Such as *EDN3*, an endothelium-derived vasoconstrictor peptide, which was up-regulated in the lungs of gayal, and may play an important role in enhancing pulmonary vasoconstrictor response (Masaki, 2000). *GJA5* is the predominant gap junction protein present in vascular endothelium, plays an important role in coupling between cells in the vascular wall (Ebong et al., 2006). *CCDC39* was related to lung development (Blanchon et al., 2012). Therefore, these genes related to promoting cardiac blood circulation and pulmonary vasoconstriction may play crucial roles in the environmental adaptability of gayal and yak.

## Discussion

In this study, we explored the high-altitude adaptability of Bovidae using the transcriptome data of six tissues from gayal and published data on yak and lowland cattle (Tang et al., 2017). Compared with numerous previous studies that only used the same reference genomes (yak or cattle genome) for different bovine subfamily species (Tang et al., 2017; Lan et al., 2018; Xin et al., 2019; Wang et al., 2020), here we optimized the experimental design, using the corresponding reference genomes for three species to reduce the gene sequence differences of different species due to phylogenetic evolution, and only retained reads that mapped to the 1:1 orthologous gene which had high-confidence alignments across the three genomes for subsequent analysis. To reduce the difference in the sequencing depth of transcripts from different sequencing platforms, we used the TMM standardized method of edgeR for correction with reference to the method of Blake et al. (Blake et al., 2020). Consistent with previous studies (Necsulea and Kaessmann, 2014; Tang et al., 2017; Hao et al., 2019), we found that the differences in global gene expression among tissues were more significant than that among altitudes or the same tissue of different species. Among them, four tissues (lung, liver, spleen, and kidney) showed tissue-specific expression patterns, while bovine species-specific expression patterns were shown in the tissue of heart and muscle (Necsulea and Kaessmann, 2014; Tang et al., 2017; Hao et al., 2019). In addition, we identified numerous DEGs in gayal and yak compared with low-altitude cattle, which might have an important role in high altitude adaptation. WGCNA was further used to explore the core genes of each tissue that regulate tissue function in the high-altitude adaptation.

### Expression Regulation of Genes Involved in the Regulation of Blood Cell Development

Hypoxia is one of the most serious challenges faced by high-altitude animals, and oxygen supply is directly related to the development of red blood cells (Zhang D. et al., 2017). Studies in mice (Li et al., 2011) and yak (Xin et al., 2019) have shown an increase in the number of red blood cells and platelets under hypoxia, but the number of granulocyte-macrophage progenitor cells periodically declined. In this study, similar results were found in the gayal. The hematopoietic cell lineage pathway was uniquely enriched in multiple tissues (heart, kidney, lung, and spleen) of gayal *vs.* the cattle group. Among these, *IL11RA* is a member of the hematopoietic cytokine receptor family (Ng et al., 2021), which was uniquely up-regulated by 1.33 times in the heart of gayal. *GP1BA* is the alpha subunit of platelet surface membrane glycoprotein Ib (Luo et al., 2007), were 9.58, 3.90, 7.77, 11.41, 12.38, 12.39-fold higher in the tissues (corresponding to heart, spleen, lung, kidney) of gayal than in the cattle, suggesting that the number of red blood cells and platelets might be increased. As for the regulatory mechanisms underlying granulocyte-macrophage progenitor cells, we observed three genes (*IL1B, CSF1, and IL6R*) were down-regulated in multi tissues of gayal, which might be a reason for the decreased proportion of granulocytes. Among them, *IL1B* is produced by activated macrophages as a proprotein (Lappas, 2013), which was down-regulated 1.94-fold in the lung of gayal. *CSF1* controls the production, differentiation, and function of macrophages (Hamilton et al., 2016), displaying log_2_ fold changes of −1.06, −1.10, and −1.76 in the heart, spleen, and muscle, respectively. In addition, Interleukin 6 is involved in the maturation of B cells. *IL6R* encodes a subunit of the interleukin 6 (*IL6*) receptor complex (Liu et al., 2017) and was found down-regulated in all tissues of gayal, which suggests lymphocytes may decrease, which is consistent with the previous results on the hypoxia adaptation of yak (Xin et al., 2019).

In addition, in the high-altitude related gene set that we searched (Supplementary Table S5), several crucial positive selection genes related to erythrocyte development were found differential expression in the gayal and yak. *EGLN1,* a key positive selection gene in the HIF pathway (Bigham et al., 2010), was found consistently down-regulated in all tissues of gayal, which contribute to enhancing HIF1-*α* activity and has a role in increased Hb levels via erythropoiesis under hypoxia conditions (Epstein et al., 2001; Xiang et al., 2013). *HFE* is a positively selected gene in Tibetans (Yi et al., 2010), which indirectly regulates iron balance by reducing the entry of transferrin into cells (Salter-Cid et al., 1999). Compared with lowland cattle, the appropriate down-regulation of *HFE* gene expression in gayal can accelerate iron storage to facilitate the synthesis of hemoglobin to cope with the reduced oxygen partial pressure (Muckenthaler et al., 2020). In the high-altitude related DEG set of yak, we also found *EGLN1* was consistently down-regulated in all tissues of yak. In addition, *HOXB6* is considered to be a marker for erythropoiesis (Magli et al., 1997), and its expression was uniquely up-regulated 1.85 and 1.11-fold in the spleen and kidney of the yak, respectively, which may contribute to yak adaptation to the hypoxia environment (Xin et al., 2019).

### Expression Regulation of Genes Involved in Angiogenesis

The regulation of angiogenesis by hypoxia links blood oxygen supply to metabolism (Pugh and Ratcliffe, 2003). In this study, the shared and unique DEGs involved in blood vessel and vascular development were identified. For example, glutamyl aminopeptidase (*ENPEP*), a member of the M1 family of endopeptidases involved in blood pressure regulation and blood vessel formation (Holmes et al., 2017), was up-regulated in the lungs of gayal and yak with the log_2_ fold changes of 2.78 and 2.98, respectively. Additionally, we found that *SRPX2* and *ESM1* were up-regulated in the spleen of gayal and yak relative to lowland cattle, and *SAT1* and *SERPINE1* genes were up-regulated in the kidney and muscle of gayal and yak relative to lowland cattle, respectively, which are angiogenesis promoting genes (Aitkenhead et al., 2002; Mohr et al., 2017; Xiao et al., 2020). *HEY1* is the downstream effector of Notch signaling required for cardiovascular development (Aitkenhead et al., 2002) with log_2_ fold changes of 1.08 and 1.71 in the heart and lung of gayal, respectively, while displaying log_2_ fold changes of 1.25 and 1.03 in the yak, respectively. In addition, *MEOX2* has been reported to have a role in inhibiting angiogenesis (Dhahri et al., 2020), and was down-regulated in all 6 tissues of gayal and the kidney of yak. Research has found that VHL-deficient mice show increased vasculogenesis in embryonic cells (Wang et al., 2017), and the *VHL* gene was down-regulated in expression in all six tissues of yak and down-regulated in the lung of gayal. In addition, we have identified two unique DEGs (*RAMP2* and *TGFBI*) in gayal that promote angiogenesis. *RAMP2* mediates the pro-angiogenic effect (Albertin et al., 2010), which was up-regulated in all six tissues of gayal. *TGFBI* is a matricellular protein-coding gene that plays an important role in tumor angiogenesis (Fico and Santamaria-Martínez, 2020), which was up-regulated in the liver and kidney of gayal with log_2_ fold changes of 1.34 and 1.45, respectively. Additionally, DEGs (*THY1* and *PLCD3*) that promote angiogenesis were also uniquely found in yak. *THY1* was expressed on mouse tumor-associated lymphatic vessels and blood vessels (Jurisic et al., 2010), displaying log_2_ fold changes of 1.63 and 1.03 in the spleen and lung of yak. *PLCD3* induce angiogenesis in human endothelium (Kim et al., 2002), which was up-regulated in all six tissues, displaying log_2_ fold changes of 1.77, 1.83, 2.07, 3.20, 2.12, and 1.15 in the heart, liver, spleen, lung, kidney, and muscle of yak, respectively. This suggests that angiogenesis may be conducive to the adaptation of gayal and yak to a high-altitude environment.

### Expression Regulation of Genes Involved in the Immune System

Increasing evidence shows that the harsh environment of hypoxia may regulate the immune system (Mishra and Ganju, 2010; Gaur et al., 2020). In the present study, we found that the immune system of the spleen in gayal and yak was highly regulated. DEGs of the spleen in gayal were significantly enriched in lymphocytic chemotaxis, inflammatory response, and cytokine receptor interaction pathways (Supplementary Table S4), and the DEGs of the spleen in yak were also significantly enriched in TNF signaling pathway, cytokine receptor interaction, and chemokine signaling pathway (Supplementary Table S4). In the spleen of gayal *vs.* cattle group, chemokines and their receptors like *CCL4, CCL5, CCL14, CCL16,* and *CXCR4* displayed log_2_ fold changes of 2.33, 2.78, 1.24, 1.83, and 1.18, respectively, which mediates chemokinetic inflammatory response (Loetscher et al., 1994; Ariel et al., 2000); Interleukins like *IL12RB2* promotes the proliferation of T-cells as well as NK cells (Presky et al., 1996), *IL23A* contribute to the production of proinflammatory cytokines (Hoeve et al., 2006), and *IL12RB2* and *IL23A* with log_2_ fold changes of 1.77 and 1.09, respectively; tumor necrosis factors like *TNFRSF8, TNFRSF4,* and *LTBR* displayed log_2_ fold changes of 4.06, 1.41, and 1.32, respectively, which activated T and B cell expression (Nishikori et al., 2005). *CD40LG* regulates B cell function by engaging *CD40* on the B cell surface (Henn et al., 1998), which was up-regulated 2.63-fold in the spleen of gayal. In the spleen of the yak *vs.* cattle group, *CXCR4* and *CD40LG* were also up-regulated, with log_2_ fold changes of 1.52 and 2.83, respectively. *CXCL14* which fulfills a unique role in antimicrobial immunity was up-regulated 2.15-fold in the spleen of yak (Maerki et al., 2009). These changes might help gayal and yak resist inflammation and disease in high-altitude harsh environments.

High altitude pulmonary edema (HAPE) is a serious life-threatening disease in humans and animals characterized by uneven vasoconstriction of pulmonary blood vessels due to hypoxia (Stream and Grissom, 2008). It has been confirmed that HAPE is related to coagulation activation and fibrin formation enhancement (Mannucci et al., 2002). In addition, hypoxia can lead to increased platelet activity, significantly increasing the risk of thrombosis (Shilei et al., 2016). In this study, we found that a large number of DEGs are related to platelet activation and blood coagulation in the two comparison groups. Coagulation factors play an important role in the endogenous and exogenous blood coagulation process in animals (Thiel et al., 2017). In this study, compared with low-altitude cattle, the expression levels of coagulation factor II (*F2*) and coagulation factor V (*F5*) in the lung of gayal were separately reduced 1.26 and 1.64-fold, and displayed log_2_ fold changes of −2.33 and −1.23 in the lung of yak, respectively. In addition, coagulation factor X (*F10*) was found uniquely down-regulated 5.62 times in the lungs of yak. Meanwhile, serine protease inhibitors are inhibitors of blood coagulation factors (Seixas et al., 2007), and we have found the expression level of *SERPINA5, SERPINF2* and *SERPING1* were uniquely increased in the lung of gayal, and the corresponding log_2_ fold changes of 1.80, 3.06, and 1.48, which may play an important role in the mechanism of pulmonary edema resistance, thus reducing the risk of pulmonary edema. In addition, impaired decomposition of bradykinin and its metabolites may be an important cause of pulmonary edema (De Maat et al., 2020). We found that the expression of the kininogen 1 (*KNG1*) gene was down-regulated by 2.46 times in the lung of yak, while down-regulated by 12.39 and 7.38-fold in the heart of gayal and yak than that of lowland cattle, respectively. These results suggest yak and gayal have developed a mechanism to prevent the occurrence of severe pulmonary edema.

### Expression Regulation of Genes Involved in Energy Metabolism

High energy metabolism or reduced energy turnover is essential for survival under hypoxia (Qiu et al., 2012). In this study, mitochondrion-related categories were widely enriched in the tissues of gayal and yak (Supplementary Table S4). Mitochondria is the main place for aerobic respiration of cells, which provides ATP for organisms through the oxidation of sugar, fats, and amino acids (Solaini et al., 2010). Similarly, several pathways related to carbohydrate and fat energy metabolism were enriched, such as glycolysis/gluconeogenesis in yak lung, oxidative phosphorylation, and galactose metabolism in multiple tissues of gayal, and PPAR signaling pathway in the heart of gayal and yak. Among the genes related to these functions, *TPI1* is essential for efficient energy production and plays an important role in glycolysis (Shimoda et al., 2012). *G6PC* (glucose-6-phosphatase) is a key enzyme of gluconeogenesis (Jia et al., 2012). We found that the gene expression of *TPI1* was up-regulated in all tissues of both gayal and yak, and *G6PC* was down-regulated in three tissues (heart, spleen, and lung) of both gayal and yak, which is conducive to the production of ATP through strengthened glycolysis and reduced gluconeogenesis. Oxidative phosphorylation is an important process for ATP syntesis in aerobic organisms. We found that most genes related to oxidative phosphorylation were up-regulated in gayal, such as *COX6A2, NDUFA9, COX8B, NDUFB7, NDUFA11, NDUFB5, UQCR10, NDUFS8, UQCRQ, PPA1,* and *NDUFS3*. In addition, the significant down-regulation of *COX5B* found in all tissues of gayal and yak may be beneficial to hypoxia adaptation (Trueblood et al., 1988). Additionally, galactose metabolism (bta00052) was uniquely enriched in the tissues (lung, liver, spleen, and muscle) of gayal. Galactose is a kind of monosaccharide, most of which are converted into glucose in the liver, and then incorporated into glycogen or used for energy metabolism by glycolysis. Lactase (*LCT*), a key enzyme involved in the conversion of galactose to glucose was up-regulated in all six tissues of gayal. The PPAR signaling pathway, a classic pathway in lipid catabolism was significantly enriched in the heart of gayal and yak. *EHHADH*, a dehydrogenase in the β-oxidase system (Yeh et al., 2006), was down-regulated 4.02 and 2.69 times in the heart of gayal and yaks. Additionally, the utilization of fatty acids first needs to be activated to produce fatty acyl CoA under the catalysis of the ACSL and SLC27A family (Bowman et al., 2016). In this study, we found that *SLC27A2* and *SLC27A6* of the SLC27A gene family were down-regulated 6.24 and 3.24 times in the heart of gayal, and *SLC27A2* and *SLC27A4* were down-regulated 6.81 and 2.08 times in yak heart, respectively. And the *ACSL3* and *ACSL4* genes expression of ACSL gene family members were found uniquely decreased in the heart of gayal. Therefore, our results suggest gayal and yak may adapt to high altitude environments by strengthening carbohydrate metabolism and reducing fatty acid degradation although the related regulation genes may be different between gayal and yak.

Despite the fact that we found numerous genes that may be related to the high-altitude adaptation of gayal and yak, we must admit that the limitation of age differences between gayal and other species, the gayal being a younger species, may affect the experimental results obtained to some extent. Therefore, it is necessary to conduct comparative transcriptome analysis on more bovine species of the same age that are combined with physiologic experiments to verify our findings, and better clarify the high-altitude adaptation mechanism of bovine subfamily species.

## Conclusion

This study shows a comprehensive multi tissues transcriptome expression landscape of high- and low-altitude bovine populations and reveals the gene expression regulation associated with high-altitude acclimatization. The gene expression profiles of gayal, yak, and cattle showed tissue-specific expression patterns. The comparative analysis of six hypoxia-related tissues of high- and low-altitude bovine subfamily species highlights numerous DEGs and terms underlying associated with hypoxia. Notably, categories related to angiogenesis, blood coagulation, energy metabolism, and the immune system were commonly enriched in the tissue DEGs of gayal *vs.* cattle group and yak *vs.* cattle group, and we found that many expression regulatory genes related to these functions in gayal and yak are different, which may serve as an important regulatory mechanism for gayal and yak to adapt to the corresponding local environment. In addition, we also found numerous hub genes related to myocardial contraction, energy metabolism, and pulmonary vasoconstriction by WGCNA analysis. Overall, our study lays a foundation for further study on the environmental adaptability of mammals in subtropical plateau and provides a valuable basis for resource utilization in high-altitude areas.

## Data Availability

The datasets presented in this study can be found in online repositories. The names of the repository/repositories and accession number(s) can be found below: NCBI PRJNA783860.

## References

[B1] Aitkenhead M. Wang S.-J. Nakatsu M. N. Mestas J. Heard C. Hughes C. C. W. (2002). Identification of Endothelial Cell Genes Expressed in an *In Vitro* Model of Angiogenesis: Induction of ESM-1, βig-h3, and NrCAM. Microvasc. Res. 63, 159–171. 10.1006/mvre.2001.2380 11866539

[B2] Anderson K. M. Anderson D. M. Mcanally J. R. Shelton J. M. Bassel-Duby R. Olson E. N. (2016). Transcription of the Non-coding RNA Upperhand Controls Hand2 Expression and Heart Development. Nature 539, 433–436. 10.1038/nature20128 27783597 PMC5261552

[B3] Ariel A. Lider O. Brill A. Cahalon L. Savion N. Varon D. (2000). Induction of Interactions between CD44 and Hyaluronic Acid by a Short Exposure of Human T Cells to Diverse Pro-inflammatory Mediators. Immunology 100, 345–351. 10.1046/j.1365-2567.2000.00059.x 10929056 PMC2327018

[B4] Bigham A. Bauchet M. Pinto D. Mao X. Akey J. M. Mei R. (2010). Identifying Signatures of Natural Selection in Tibetan and Andean Populations Using Dense Genome Scan Data. Plos Genet. 6, e1001116. 10.1371/journal.pgen.1001116 20838600 PMC2936536

[B5] Blake L. E. Roux J. Hernando-Herraez I. Banovich N. E. Perez R. G. Hsiao C. J. (2020). A Comparison of Gene Expression and DNA Methylation Patterns across Tissues and Species. Genome Res. 30, 250–262. 10.1101/gr.254904.119 31953346 PMC7050529

[B6] Blanchon S. Legendre M. Copin B. Duquesnoy P. Montantin G. Kott E. (2012). Delineation ofCCDC39/CCDC40mutation Spectrum and Associated Phenotypes in Primary Ciliary Dyskinesia. J. Med. Genet. 49, 410–416. 10.1136/jmedgenet-2012-100867 22693285

[B7] Bowman T. A. O'keeffe K. R. D'aquila T. Yan Q. W. Griffin J. D. Killion E. A. (2016). Acyl CoA Synthetase 5 (ACSL5) Ablation in Mice Increases Energy Expenditure and Insulin Sensitivity and Delays Fat Absorption. Mol. Metab. 5, 210–220. 10.1016/j.molmet.2016.01.001 26977393 PMC4770262

[B8] Camacho C. Coulouris G. Avagyan V. Ma N. Papadopoulos J. Bealer K. (2009). BLAST+: Architecture and Applications. BMC Bioinformatics 10, 421. 10.1186/1471-2105-10-421 20003500 PMC2803857

[B9] Chen S. Yang D. Lei C. Li Y. Sun X. Chen M. (2019). Identification of Crucial Genes in Abdominal Aortic Aneurysm by WGCNA. PeerJ 7, e7873. 10.7717/peerj.7873 31608184 PMC6788446

[B10] Conway J. R. Lex A. Gehlenborg N. (2017). UpSetR: an R Package for the Visualization of Intersecting Sets and Their Properties. Bioinformatics 33, 2938–2940. 10.1093/bioinformatics/btx364 28645171 PMC5870712

[B11] De Maat S. De Mast Q. Danser A. H. J. Van De Veerdonk F. L. Maas C. (2020). Impaired Breakdown of Bradykinin and its Metabolites as a Possible Cause for Pulmonary Edema in COVID-19 Infection. Semin. Thromb. Hemost. 46, 835–837. 10.1055/s-0040-1712960 32526773 PMC7645818

[B12] Dhahri W. Dussault S. Légaré É. Rivard F. Desjarlais M. Mathieu R. (2020). Reduced Expression of microRNA-130a Promotes Endothelial Cell Senescence and Age-dependent Impairment of Neovascularization. Aging 12, 10180–10193. 10.18632/aging.103340 32457253 PMC7346016

[B13] Duquesnoy P. Escudier E. Vincensini L. Freshour J. Bridoux A.-M. Coste A. (2009). Loss-of-function Mutations in the Human Ortholog of Chlamydomonas Reinhardtii ODA7 Disrupt Dynein Arm Assembly and Cause Primary Ciliary Dyskinesia. Am. J. Hum. Genet. 85, 890–896. 10.1016/j.ajhg.2009.11.008 19944405 PMC2790569

[B14] Ebong E. E. Kim S. Depaola N. (2006). Flow Regulates Intercellular Communication in HAEC by Assembling Functional Cx40 and Cx37 gap Junctional Channels. Am. J. Physiology-Heart Circulatory Physiol. 290, H2015–H2023. 10.1152/ajpheart.00204.2005 16361362

[B15] Emms D. M. Kelly S. (2015). OrthoFinder: Solving Fundamental Biases in Whole Genome Comparisons Dramatically Improves Orthogroup Inference Accuracy. Genome Biol. 16, 157. 10.1186/s13059-015-0721-2 26243257 PMC4531804

[B16] Epstein A. C. R. Gleadle J. M. Mcneill L. A. Hewitson K. S. O'rourke J. Mole D. R. (2001). *C. elegans* EGL-9 and Mammalian Homologs Define a Family of Dioxygenases that Regulate HIF by Prolyl Hydroxylation. Cell 107, 43–54. 10.1016/s0092-8674(01)00507-4 11595184

[B17] Erginel-Unaltuna N. Yang W.-P. Blanar M. A. (1998). Genomic Organization and Expression of KCNJ8/Kir6.1, a Gene Encoding a Subunit of an ATP-Sensitive Potassium Channel. Gene 211, 71–78. 10.1016/s0378-1119(98)00086-9 9573340

[B18] Feng S. Ma J. Long K. Zhang J. Qiu W. Li Y. (2020). Comparative microRNA Transcriptomes in Domestic Goats Reveal Acclimatization to High Altitude. Front. Genet. 11, 809. 10.3389/fgene.2020.00809 32849809 PMC7411263

[B19] Fernández-Vizarra E. Zeviani M. (2015). Nuclear Gene Mutations as the Cause of Mitochondrial Complex III Deficiency. Front. Genet. 6, 1. 10.3389/fgene.2015.00134 25914718 PMC4391031

[B20] Fico F. Santamaria‐Martínez A. (2020). TGFBI Modulates Tumour Hypoxia and Promotes Breast Cancer Metastasis. Mol. Oncol. 14, 3198–3210. 10.1002/1878-0261.12828 33080107 PMC7718944

[B21] Gaur P. Saini S. Ray K. Asanbekovna K. N. Akunov A. Maripov A. (2020). Temporal Transcriptome Analysis Suggest Modulation of Multiple Pathways and Gene Network Involved in Cell-Cell Interaction during Early Phase of High Altitude Exposure. PLoS One 15, e0238117. 10.1371/journal.pone.0238117 32911517 PMC7482924

[B22] Ge R.-L. Simonson T. S. Cooksey R. C. Tanna U. Qin G. Huff C. D. (2012). Metabolic Insight into Mechanisms of High-Altitude Adaptation in Tibetans. Mol. Genet. Metab. 106, 244–247. 10.1016/j.ymgme.2012.03.003 22503288 PMC3437309

[B23] Gou W. Peng J. Wu Q. Zhang Q. Zhang H. Wu C. (2014). Expression Pattern of Heme Oxygenase 1 Gene and Hypoxic Adaptation in Chicken Embryos. Comp. Biochem. Physiol. B: Biochem. Mol. Biol. 174, 23–28. 10.1016/j.cbpb.2014.05.005 24947210

[B24] Guidolin G. Sorato E. Oselladore B. Mascarin A. Tortorella C. Guidolin D. (2010). Involvement of Vascular Endothelial Growth Factor Signaling in CLR/RAMP1 and CLR/RAMP2-mediated Pro-angiogenic Effect of Intermedin on Human Vascular Endothelial Cells. Int. J. Mol. Med. 26, 289–294. 10.3892/ijmm_00000464 20596610

[B25] Haasl R. J. Payseur B. A. (2016). Fifteen Years of Genomewide Scans for Selection: Trends, Lessons and Unaddressed Genetic Sources of Complication. Mol. Ecol. 25, 5–23. 10.1111/mec.13339 26224644 PMC4868130

[B26] Hamilton J. A. Cook A. D. Tak P. P. (2016). Anti-colony-stimulating Factor Therapies for Inflammatory and Autoimmune Diseases. Nat. Rev. Drug Discov. 16, 53–70. 10.1038/nrd.2016.231 28031576

[B27] Hao Y. Xiong Y. Cheng Y. Song G. Jia C. Qu Y. (2019). Comparative Transcriptomics of 3 High-Altitude Passerine Birds and Their Low-Altitude Relatives. Proc. Natl. Acad. Sci. USA 116, 11851–11856. 10.1073/pnas.1819657116 31127049 PMC6576129

[B28] Hautala A. J. Rankinen T. Kiviniemi A. M. Mäkikallio T. H. Huikuri H. V. Bouchard C. (2006). Heart Rate Recovery after Maximal Exercise Is Associated with Acetylcholine Receptor M2 (CHRM2) Gene Polymorphism. Am. J. Physiology-Heart Circulatory Physiol. 291, H459–H466. 10.1152/ajpheart.01193.2005 16501017

[B29] Heinrich E. C. Wu L. Lawrence E. S. Cole A. M. Anza‐Ramirez C. Villafuerte F. C. (2019). Genetic Variants at the EGLN1 Locus Associated with High‐altitude Adaptation in Tibetans Are Absent or Found at Low Frequency in highland Andeans. Ann. Hum. Genet. 83, 171–176. 10.1111/ahg.12299 30719713 PMC7920394

[B30] Henn V. Slupsky J. R. Gräfe M. Anagnostopoulos I. Förster R. Müller-Berghaus G. (1998). CD40 Ligand on Activated Platelets Triggers an Inflammatory Reaction of Endothelial Cells. Nature 391, 591–594. 10.1038/35393 9468137

[B31] Hoeve M. A. Savage N. D. L. De Boer T. Langenberg D. M. L. de Waal Malefyt R. Ottenhoff T. H. M. (2006). Divergent Effects of IL-12 and IL-23 on the Production of IL-17 by Human T Cells. Eur. J. Immunol. 36, 661–670. 10.1002/eji.200535239 16482511

[B32] Holmes R. S. Spradling Reeves K. D. Cox L. A. (2017). Mammalian Glutamyl Aminopeptidase Genes (ENPEP) and Proteins: Comparative Studies of a Major Contributor to Arterial Hypertension. J. Data Mining Genomics Proteomics 08. 10.4172/2153-0602.1000211 PMC5995572 29900035

[B33] Huang D. W. Sherman B. T. Lempicki R. A. (2009). Systematic and Integrative Analysis of Large Gene Lists Using DAVID Bioinformatics Resources. Nat. Protoc. 4, 44–57. 10.1038/nprot.2008.211 19131956

[B34] Huang Y. Li S.-n. Zhou X.-y. Zhang L.-x. Chen G.-x. Wang T.-h. (2019). The Dual Role of AQP4 in Cytotoxic and Vasogenic Edema Following Spinal Cord Contusion and its Possible Association with Energy Metabolism via COX5A. Front. Neurosci. 13, 584. 10.3389/fnins.2019.00584 31258460 PMC6587679

[B35] Jia C. Kong X. Koltes J. E. Gou X. Yang S. Yan D. (2016). Gene Co-expression Network Analysis Unraveling Transcriptional Regulation of High-Altitude Adaptation of Tibetan Pig. PLoS One 11, e0168161. 10.1371/journal.pone.0168161 27936142 PMC5148111

[B36] Jia Y. Cong R. Li R. Yang X. Sun Q. Parvizi N. (2012). Maternal Low-Protein Diet Induces Gender-dependent Changes in Epigenetic Regulation of the Glucose-6-Phosphatase Gene in Newborn Piglet Liver. J. Nutr. 142, 1659–1665. 10.3945/jn.112.160341 22833655

[B37] Jurisic G. Iolyeva M. Proulx S. T. Halin C. Detmar M. (2010). Thymus Cell Antigen 1 (Thy1, CD90) Is Expressed by Lymphatic Vessels and Mediates Cell Adhesion to Lymphatic Endothelium. Exp. Cel Res. 316, 2982–2992. 10.1016/j.yexcr.2010.06.013 PMC3398154 20599951

[B38] Kaiser R. Friedrich D. Chavakis E. Böhm M. Friedrich E. B. (2012). Effect of Hypoxia on Integrin-Mediated Adhesion of Endothelial Progenitor Cells. J. Cel. Mol. Med. 16, 2387–2393. 10.1111/j.1582-4934.2012.01553.x PMC3823432 22353471

[B39] Keren L. Ke M. Tiandong C. Jinwei Z. Wanling Q. Yujie W. (2018). Transcriptome Differences in Frontal Cortex between Wild Boar and Domesticated Pig. Anim. Sci. J. = Nihon chikusan Gakkaiho 89. 10.1111/asj.12999 29536589

[B40] Kim Y.-M. Kim Y.-M. Lee Y. M. Kim H.-S. Kim J. D. Choi Y. (2002). TNF-related Activation-Induced Cytokine (TRANCE) Induces Angiogenesis through the Activation of Src and Phospholipase C (PLC) in Human Endothelial Cells. J. Biol. Chem. 277, 6799–6805. 10.1074/jbc.M109434200 11741951

[B41] Lan D. Xiong X. Ji W. Li J. Mipam T.-D. Ai Y. (2018). Transcriptome Profile and Unique Genetic Evolution of Positively Selected Genes in Yak Lungs. Genetica 146, 151–160. 10.1007/s10709-017-0005-8 29285685

[B42] Langfelder P. Horvath S. (2008). WGCNA: an R Package for Weighted Correlation Network Analysis. BMC Bioinformatics 9, 559. 10.1186/1471-2105-9-559 19114008 PMC2631488

[B43] Lappas M. (2013). NOD1 and NOD2 Regulate Proinflammatory and Prolabor Mediators in Human Fetal Membranes and Myometrium via Nuclear Factor-Kappa B1. Biol. Reprod. 89, 14. 10.1095/biolreprod.113.110056 23740944

[B44] Lemieux H. Hoppel C. L. (2009). Mitochondria in the Human Heart. J. Bioenerg. Biomembr 41, 99–106. 10.1007/s10863-009-9211-0 19353253

[B45] Li P. Huang J. Tian H.-j. Huang Q.-y. Jiang C.-h. Gao Y.-q. (2011). Regulation of Bone Marrow Hematopoietic Stem Cell Is Involved in High-Altitude Erythrocytosis. Exp. Hematol. 39, 37–46. 10.1016/j.exphem.2010.10.006 20977927

[B46] Liao Y. Smyth G. K. Shi W. (2014). featureCounts: an Efficient General Purpose Program for Assigning Sequence Reads to Genomic Features. Bioinformatics 30, 923–930. 10.1093/bioinformatics/btt656 24227677

[B47] Liu A. Wang Y. Sahana G. Zhang Q. Liu L. Lund M. S. (2017). Genome-wide Association Studies for Female Fertility Traits in Chinese and Nordic Holsteins. Sci. Rep. 7, 8487. 10.1038/s41598-017-09170-9 28814769 PMC5559619

[B48] Loetscher M. Geiser T. O'reilly T. Zwahlen R. Baggiolini M. Moser B. (1994). Cloning of a Human Seven-Transmembrane Domain Receptor, LESTR, that Is Highly Expressed in Leukocytes. J. Biol. Chem. 269, 232–237. 10.1016/s0021-9258(17)42339-8 8276799

[B49] Luo S.-Z. Mo X. Afshar-Kharghan V. Srinivasan S. López J. A. Li R. (2007). Glycoprotein Ibα Forms Disulfide Bonds with 2 Glycoprotein Ibβ Subunits in the Resting Platelet. Blood 109, 603–609. 10.1182/blood-2006-05-024091 17008541 PMC1785083

[B50] Luo Y. Wang X. Ma L. Ma Z. Li S. Fang X. (2020). Bioinformatics Analyses and Biological Function of lncRNA ZFPM2-AS1 and ZFPM2 G-ene in H-epatocellular C-arcinoma. Oncol. Lett. 19, 3677–3686. 10.3892/ol.2020.11485 32382322 PMC7202276

[B51] Maerki C. Meuter S. Liebi M. Mühlemann K. Frederick M. J. Yawalkar N. (2009). Potent and Broad-Spectrum Antimicrobial Activity of CXCL14 Suggests an Immediate Role in Skin Infections. J. Immunol. 182, 507–514. 10.4049/jimmunol.182.1.507 19109182

[B52] Magli M. C. Largman C. Lawrence H. J. (1997). Effects ofHOX Homeobox Genes in Blood Cell Differentiation. J. Cel. Physiol. 173, 168–177. 10.1002/(sici)1097-4652(199711)173:2<168::aid-jcp16>3.0.co;2-c 9365517

[B53] Mannucci P. M. Gringeri A. Peyvandi F. Di Paolantonio T. Mariani G. (2002). Short-term Exposure to High Altitude Causes Coagulation Activation and Inhibits Fibrinolysis. Thromb. Haemost. 87, 342–343. 11858498

[B54] Masaki T. (2000). The Endothelin Family: an Overview. J. Cardiovasc. Pharmacol. 35, S3–S5. 10.1097/00005344-200000002-00002 10976772

[B55] Merkin J. Russell C. Chen P. Burge C. B. (2012). Evolutionary Dynamics of Gene and Isoform Regulation in Mammalian Tissues. Science 338, 1593–1599. 10.1126/science.1228186 23258891 PMC3568499

[B56] Miao F. Guo Z. Xue R. Wang X. Shen Y. (2015). Effects of Grazing and Precipitation on Herbage Biomass, Herbage Nutritive Value, and Yak Performance in an Alpine Meadow on the Qinghai-Tibetan Plateau. Plos One 10, e0127275. 10.1371/journal.pone.0127275 26039322 PMC4454548

[B57] Mishra K. P. Ganju L. (2010). Influence of High Altitude Exposure on the Immune System: a Review. Immunological Invest. 39, 219–234. 10.3109/08820131003681144 20380520

[B58] Mohr T. Haudek-Prinz V. Slany A. Grillari J. Micksche M. Gerner C. (2017). Proteome Profiling in IL-1β and VEGF-Activated Human Umbilical Vein Endothelial Cells Delineates the Interlink between Inflammation and Angiogenesis. PLoS One 12, e0179065. 10.1371/journal.pone.0179065 28617818 PMC5472280

[B59] Mortazavi A. Williams B. A. Mccue K. Schaeffer L. Wold B. (2008). Mapping and Quantifying Mammalian Transcriptomes by RNA-Seq. Nat. Methods 5, 621–628. 10.1038/nmeth.1226 18516045 PMC13303166

[B60] Muckenthaler M. U. Mairbäurl H. Gassmann M. (20201985). Iron Metabolism in High-Altitude Residents. J. Appl. Physiol. 129, 920–925. 10.1152/japplphysiol.00019.2020 32853112

[B61] Mukherjee S. Mukherjee A. Jasrotia R. S. Jaiswal S. Iquebal M. A. Longkumer I. (2020). Muscle Transcriptome Signature and Gene Regulatory Network Analysis in Two Divergent Lines of a Hilly Bovine Species Mithun (*Bos frontalis*). Genomics 112, 252–262. 10.1016/j.ygeno.2019.02.004 30822468

[B62] Necsulea A. Kaessmann H. (2014). Evolutionary Dynamics of Coding and Non-coding Transcriptomes. Nat. Rev. Genet. 15, 734–748. 10.1038/nrg3802 25297727

[B63] Ng B. Widjaja A. A. Viswanathan S. Dong J. Chothani S. P. Lim S. (2021). Similarities and Differences between IL11 and IL11RA1 Knockout Mice for Lung Fibro-Inflammation, Fertility and Craniosynostosis. Sci. Rep. 11, 14088. 10.1038/s41598-021-93623-9 34239012 PMC8266813

[B64] Nishikori M. Ohno H. Haga H. Uchiyama T. (2005). Stimulation of CD30 in Anaplastic Large Cell Lymphoma Leads to Production of Nuclear Factor-kappaB P52, Which Is Associated with Hyperphosphorylated Bcl-3. Cancer Sci. 96, 487–497. 10.1111/j.1349-7006.2005.00078.x 16108830 PMC11159099

[B65] Ozsolak F. Milos P. M. (2011). RNA Sequencing: Advances, Challenges and Opportunities. Nat. Rev. Genet. 12, 87–98. 10.1038/nrg2934 21191423 PMC3031867

[B66] Pamenter M. E. Hall J. E. Tanabe Y. Simonson T. S. (2020). Cross-Species Insights into Genomic Adaptations to Hypoxia. Front. Genet. 11, 1. 10.3389/fgene.2020.00743 32849780 PMC7387696

[B67] Pan H. Xiang H. Wang J. Wei Z. Zhou Y. Liu B. (2019). CAPS Mutations Are Potentially Associated with Unexplained Recurrent Pregnancy Loss. Am. J. Pathol. 189, 124–131. 10.1016/j.ajpath.2018.09.010 30339840

[B68] Peng Y. Cui C. He Y. OuzhuluobuZhang H. Zhang H. Yang D. (2017). Down-Regulation ofEPAS1Transcription and Genetic Adaptation of Tibetans to High-Altitude Hypoxia. Mol. Biol. Evol. 34, msw280–830. 10.1093/molbev/msw280 PMC5400376 28096303

[B69] Pertea M. Kim D. Pertea G. M. Leek J. T. Salzberg S. L. (2016). Transcript-level Expression Analysis of RNA-Seq Experiments with HISAT, StringTie and Ballgown. Nat. Protoc. 11, 1650–1667. 10.1038/nprot.2016.095 27560171 PMC5032908

[B70] Presky D. H. Yang H. Minetti L. J. Chua A. O. Nabavi N. Wu C.-Y. (1996). A Functional Interleukin 12 Receptor Complex Is Composed of Two -type Cytokine Receptor Subunits. Proc. Natl. Acad. Sci. 93, 14002–14007. 10.1073/pnas.93.24.14002 8943050 PMC19484

[B71] Pugh C. W. Ratcliffe P. J. (2003). Regulation of Angiogenesis by Hypoxia: Role of the HIF System. Nat. Med. 9, 677–684. 10.1038/nm0603-677 12778166

[B72] Qi X. Zhang Q. He Y. Yang L. Zhang X. Shi P. (2019). The Transcriptomic Landscape of Yaks Reveals Molecular Pathways for High Altitude Adaptation. Genome Biol. Evol. 11, 72–85. 10.1093/gbe/evy264 30517636 PMC6320679

[B73] Qiu Q. Zhang G. Ma T. Qian W. Wang J. Ye Z. (2012). The Yak Genome and Adaptation to Life at High Altitude. Nat. Genet. 44, 946–949. 10.1038/ng.2343 22751099

[B74] Robinson M. D. Mccarthy D. J. Smyth G. K. (2010). edgeR: a Bioconductor Package for Differential Expression Analysis of Digital Gene Expression Data. Bioinformatics 26, 139–140. 10.1093/bioinformatics/btp616 19910308 PMC2796818

[B75] Salleh M. S. Mazzoni G. Höglund J. K. Olijhoek D. W. Lund P. Løvendahl P. (2017). RNA-seq Transcriptomics and Pathway Analyses Reveal Potential Regulatory Genes and Molecular Mechanisms in High- and Low-Residual Feed Intake in Nordic Dairy Cattle. BMC Genomics 18, 258. 10.1186/s12864-017-3622-9 28340555 PMC5366136

[B76] Salter-Cid L. Brunmark A. Li Y. Leturcq D. Peterson P. A. Jackson M. R. (1999). Transferrin Receptor Is Negatively Modulated by the Hemochromatosis Protein HFE: Implications for Cellular Iron Homeostasis. Proc. Natl. Acad. Sci. 96, 5434–5439. 10.1073/pnas.96.10.5434 10318901 PMC21877

[B77] Seixas S. Suriano G. Carvalho F. Seruca R. Rocha J. Di Rienzo A. (2007). Sequence Diversity at the Proximal 14q32.1 SERPIN Subcluster: Evidence for Natural Selection Favoring the Pseudogenization of SERPINA2. Mol. Biol. Evol. 24, 587–598. 10.1093/molbev/msl187 17135331

[B78] Shan L. Kawakami T. Asano S. Noritake S. Yoshimoto D. Yamashita K. (2009). Inverse Relationship between Sec14l3 mRNA/protein Expression and Allergic Airway Inflammation. Eur. J. Pharmacol. 616, 293–300. 10.1016/j.ejphar.2009.06.055 19577556

[B79] Sharma M. Singh S. B. Sarkar S. (2014). Genome Wide Expression Analysis Suggests Perturbation of Vascular Homeostasis during High Altitude Pulmonary Edema. PLoS One 9, e85902. 10.1371/journal.pone.0085902 24465776 PMC3899118

[B80] Shilei C. Changhong D. Mingqiang S. Gaomei Z. Yang X. Ke Y. (2016). Sympathetic Stimulation Facilitates Thrombopoiesis by Promoting Megakaryocyte Adhesion, Migration, and Proplatelet Formation. Blood 127. 10.1182/blood-2015-07-660746 26644453

[B81] Shimoda Y. Han J. Kawada K. Smaoui A. Isoda H. (20122012). Metabolomics Analysis ofCistus monspeliensisLeaf Extract on Energy Metabolism Activation in Human Intestinal Cells. J. Biomed. Biotechnol. 2012, 1–7. 10.1155/2012/428514 PMC3317194 22523469

[B82] Simonson T. S. Yang Y. Huff C. D. Yun H. Qin G. Witherspoon D. J. (2010). Genetic Evidence for High-Altitude Adaptation in Tibet. Science 329, 72–75. 10.1126/science.1189406 20466884

[B83] Solaini G. Baracca A. Lenaz G. Sgarbi G. (2010). Hypoxia and Mitochondrial Oxidative Metabolism. Biochim. Biophys. Acta (Bba) - Bioenerg. 1797, 1171–1177. 10.1016/j.bbabio.2010.02.011 20153717

[B84] Storz J. F. (2021). High-Altitude Adaptation: Mechanistic Insights from Integrated Genomics and Physiology. Mol. Biol. Evol. 38, 2677–2691. 10.1093/molbev/msab064 33751123 PMC8233491

[B85] Stream J. O. Grissom C. K. (2008). Update on High-Altitude Pulmonary Edema: Pathogenesis, Prevention, and Treatment. Wilderness Environ. Med. 19, 293–303. 10.1580/07-weme-rev-173.1 19099331

[B86] Sultan M. Schulz M. H. Richard H. Magen A. Klingenhoff A. Scherf M. (2008). A Global View of Gene Activity and Alternative Splicing by Deep Sequencing of the Human Transcriptome. Science 321, 956–960. 10.1126/science.1160342 18599741

[B87] Tang Q. Gu Y. Zhou X. Jin L. Guan J. Liu R. (2017). Comparative Transcriptomics of 5 High-Altitude Vertebrates and Their Low-Altitude Relatives. Gigascience 6, 1–9. 10.1093/gigascience/gix105 PMC5729692 29149296

[B88] Tashi T. Feng T. Koul P. Amaru R. Hussey D. Lorenzo F. R. (2014). High Altitude Genetic Adaptation in Tibetans: No Role of Increased Hemoglobin-Oxygen Affinity. Blood Cell Mol. Dis. 53, 27–29. 10.1016/j.bcmd.2014.02.003 PMC4039493 24618341

[B89] Thiel A. Mogel H. Bruggisser J. Baumann A. Wyder M. Stoffel M. (2017). Effect of *Clostridium perfringens* β-Toxin on Platelets. Toxins 9, 336. 10.3390/toxins9100336 PMC5666382 29064418

[B90] Tian Y. B. He S. Y. Ge C. R. (1998). Gayal. J. Yellow Cattle Sci. 6-13, 39.

[B91] Touma M. Kang X. Zhao Y. Cass A. A. Gao F. Biniwale R. (2016). Decoding the Long Noncoding RNA during Cardiac Maturation. Circ. Cardiovasc. Genet. 9, 395–407. 10.1161/circgenetics.115.001363 27591185 PMC5085833

[B92] Trueblood C. E. Wright R. M. Poyton R. O. (1988). Differential Regulation of the Two Genes Encoding *Saccharomyces cerevisiae* Cytochrome C Oxidase Subunit V by Heme and the HAP2 and REO1 Genes. Mol. Cel Biol 8, 4537–4540. 10.1128/mcb.8.10.4537-4540.1988 PMC365531 2847035

[B93] Uzzaman M. R. Bhuiyan M. S. A. Edea Z. Kim K.-S. (2014). Semi-domesticated and Irreplaceable Genetic Resource Gayal (*Bos frontalis*) Needs Effective Genetic Conservation in Bangladesh: A Review. Asian Australas. J. Anim. Sci. 27, 1368–1372. 10.5713/ajas.2014.14159 25178382 PMC4150205

[B94] Vukicevic S. Helder M. N. Luyten F. P. (1994). Developing Human Lung and Kidney Are Major Sites for Synthesis of Bone Morphogenetic Protein-3 (Osteogenin). J. Histochem. Cytochem. 42, 869–875. 10.1177/42.7.8014470 8014470

[B95] Wang J. Chai Z. Deng L. Wang J. Wang H. Tang Y. (2020). Detection and Integrated Analysis of lncRNA and mRNA Relevant to Plateau Adaptation of Yak. Reprod. Dom Anim. 55, 1461–1469. 10.1111/rda.13767 PMC7756654 32633845

[B96] Wang K. Yang Y. Wang L. Ma T. Shang H. Ding L. (2016). Different Gene Expressions between Cattle and Yak Provide Insights into High-Altitude Adaptation. Anim. Genet. 47, 28–35. 10.1111/age.12377 26538003

[B97] Wang T. Guo Y. Liu S. Zhang C. Cui T. Ding K. (2021). KLF4, a Key Regulator of a Transitive Triplet, Acts on the TGF-β Signaling Pathway and Contributes to High-Altitude Adaptation of Tibetan Pigs. Front. Genet. 12, 1. 10.3389/fgene.2021.628192 PMC8082500 33936161

[B98] Wang Y. Chen D.-Q. Chen M.-Y. Ji K.-Y. Ma D.-X. Zhou L.-F. (2017). Endothelial Cells by Inactivation of VHL Gene Direct Angiogenesis, Not Vasculogenesis via Twist1 Accumulation Associated with Hemangioblastoma Neovascularization. Sci. Rep. 7, 5463. 10.1038/s41598-017-05833-9 28710479 PMC5511164

[B99] Waypa G. B. Schumacker P. T. (2002). O2 Sensing in Hypoxic Pulmonary Vasoconstriction: the Mitochondrial Door Re-opens. Respir. Physiol. Neurobiol. 132, 81–91. 10.1016/s1569-9048(02)00051-4 12126697

[B100] Wei Q. Yu H. X. (2008). Comparison of Histological Structure of Pulmonary Alveoli between 180 Days Old Yak and plain Cattle. J. Qinghai Univ. (Natural Sci. Edition) 1, 36–39.

[B101] Winter H. Mayr B. Schleger W. Dworak E. Krutzler J. Kalat M. (1986). Genetic Characterisation of the Mithun (*Bos frontalis*) and Studies of Spermatogenesis, Blood Groups and Haemoglobins of its Hybrids with *Bos indicus* . Res. Vet. Sci. 40, 8–17. 10.1016/s0034-5288(18)30479-x 3704329

[B102] Xiang K. OuzhuluobuPeng Y. Peng Y. Yang Z. Zhang X. Cui C. (2013). Identification of a Tibetan-specific Mutation in the Hypoxic Gene EGLN1 and its Contribution to High-Altitude Adaptation. Mol. Biol. Evol. 30, 1889–1898. 10.1093/molbev/mst090 23666208

[B103] Xiao Y. Li C. Wang H. Liu Y. (2020). LINC00265 Targets miR-382-5p to Regulate SAT1, VAV3 and Angiogenesis in Osteosarcoma. aging 12, 20212–20225. 10.18632/aging.103762 33109774 PMC7655165

[B104] Xin J.-W. Chai Z.-X. Zhang C.-F. Zhang Q. Zhu Y. Cao H.-W. (2019). Transcriptome Profiles Revealed the Mechanisms Underlying the Adaptation of Yak to High-Altitude Environments. Sci. Rep. 9, 7558. 10.1038/s41598-019-43773-8 31101838 PMC6525198

[B105] Yang Y. Han L. Yuan Y. Li J. Hei N. Liang H. (2014). Gene Co-expression Network Analysis Reveals Common System-Level Properties of Prognostic Genes across Cancer Types. Nat. Commun. 5, 3231. 10.1038/ncomms4231 24488081 PMC3951205

[B106] Yeh C.-S. Wang J.-Y. Cheng T.-L. Juan C.-H. Wu C.-H. Lin S.-R. (2006). Fatty Acid Metabolism Pathway Play an Important Role in Carcinogenesis of Human Colorectal Cancers by Microarray-Bioinformatics Analysis. Cancer Lett. 233, 297–308. 10.1016/j.canlet.2005.03.050 15885896

[B107] Yi T. Wu X. Li H. (2020). Ubiquinol-cytochrome C Reductase Core Protein 1 Overexpression Protects H9c2 Cardiac Cells against Mimic Ischemia/reperfusion Injury through PI3K/Akt/GSK-3β Pathway. Biochem. Biophysical Res. Commun. 529, 904–909. 10.1016/j.bbrc.2020.06.089 32819597

[B108] Yi X. Liang Y. Huerta-Sanchez E. Jin X. Cuo Z. X. P. Pool J. E. (2010). Sequencing of 50 Human Exomes Reveals Adaptation to High Altitude. Science 329, 75–78. 10.1126/science.1190371 20595611 PMC3711608

[B109] Zhang B. Chamba Y. Shang P. Wang Z. Ma J. Wang L. (2017a). Comparative Transcriptomic and Proteomic Analyses Provide Insights into the Key Genes Involved in High-Altitude Adaptation in the Tibetan Pig. Sci. Rep. 7, 3654. 10.1038/s41598-017-03976-3 28623314 PMC5473931

[B110] Zhang C. Wang G. Wang J. Ji Z. Liu Z. Pi X. (2013). Characterization and Comparative Analyses of Muscle Transcriptomes in Dorper and Small-Tailed Han Sheep Using RNA-Seq Technique. PLoS One 8, e72686. 10.1371/journal.pone.0072686 24023632 PMC3758325

[B111] Zhang D. Yu M. Hu P. Peng S. Liu Y. Li W. (2017b). Genetic Adaptation of Schizothoracine Fish to the Phased Uplifting of the Qinghai-Tibetan Plateau. G3 (Bethesda) 7, 1267–1276. 10.1534/g3.116.038406 28209761 PMC5386875

[B112] Zhang W. Fan Z. Han E. Hou R. Zhang L. Galaverni M. (2014). Hypoxia Adaptations in the Grey Wolf (*Canis lupus* Chanco) from Qinghai-Tibet Plateau. Plos Genet. 10, e1004466. 10.1371/journal.pgen.1004466 25078401 PMC4117439

[B113] Zimmermann F. Rich I. N. (1997). Mammalian Homeobox B6 Expression Can Be Correlated with Erythropoietin Production Sites and Erythropoiesis during Development, but Not with Hematopoietic or Nonhematopoietic Stem Cell Populations. Blood 89, 2723–2735. 10.1182/blood.v89.8.2723 9108390

