# Erratum: Comparative Transcriptome Analysis of Gayal (*Bos frontalis*), Yak (*Bos grunniens*), and Cattle (*Bos taurus*) Reveal the High-Altitude Adaptation

**DOI:** 10.3389/fgene.2022.868475

**Published:** 2022-03-07

**Authors:** Frontiers Production Office

**Affiliations:** Frontiers Media SA, Lausanne, Switzerland

**Keywords:** gayal, yak, differentially expressed genes, co-expression, high-altitude adaptation, hypoxia

Due to a production error, a version of the article with grammatical mistakes was published.

The publisher apologizes for this mistake. The original version of this article has been updated.

